# Pyrolysis of Automotive Shredder Residue (ASR): Thermogravimetry, In-Situ Synchrotron IR and Gas-Phase IR of Polymeric Components

**DOI:** 10.3390/polym15173650

**Published:** 2023-09-04

**Authors:** Isha Kohli, Srikanth Chakravartula Srivatsa, Oisik Das, Sheila Devasahayam, R. K. Singh Raman, Sankar Bhattacharya

**Affiliations:** 1 Department of Chemical and Biological Engineering, Monash University, Clayton, VIC 3800, Australia; ishakohli3242@gmail.com (I.K.); srikanth.cs@monash.edu (S.C.S.); 2 Department of Civil, Environmental and Natural Resources Engineering, Luleå University of Technology, 97187 Luleå, Sweden; oisik.das@ltu.se; 3 WASM—Minerals, Energy and Chemical Engineering, Curtin University, Perth, WA 6845, Australia; sheila.devasahayam@curtin.edu.au; 4 Department of Mechanical & Aerospace Engineering, Monash University, Clayton, VIC 3800, Australia

**Keywords:** automotive shredder residue, thermogravimetry, in situ synchrotron IR, gas-phase IR, heating rates, activation energy

## Abstract

This article reports the characterisation of pyrolysis of automotive shredder residue using in situ synchrotron IR, gas-phase IR, and thermal analyses to explore if the automotive shredder residue can be converted into value-added products. When heating to ~600 °C at different heating rates, thermal analyses suggested one- to two-stage pyrolysis. Transformations in the first stage, at lower temperatures, were attributed to the degradation of carbonyl, hydroxyl, or carboxyl functional stabilisers (aldehyde and ether impurities, additives, and stabilisers in the ASR). The second stage transformations, at higher temperatures, were attributed to the thermal degradation of the polymer char. Simultaneous thermal analyses and gas-phase IR spectroscopy confirmed the evolution of the gases (alkanes (CH_4_), CO_2_, and moisture). The synchrotron IR data have demonstrated that a high heating rate (such as 150 °C/min) results in an incomplete conversion of ASRs unless sufficient time is provided. The thermogravimetry data fit the linearised multistage kinetic model at different heating rates. The activation energy of reactions varied between 24.98 and 124.94 kJ/mol, indicating a surface-controlled reaction exhibiting high activation energy during the initial stages and a diffusion and mass transfer control showing lower activation energy at the final stages. The corresponding frequency factors were in the range of 3.34 × 10^13^–5.68 × 10^1^ mg^−1^/min for different pyrolysis stages. The evolution of the functional groups decreased with an increase in the heating rate.

## 1. Introduction

It is estimated that the number of end-of-life vehicles (ELVs) worldwide is about 50 million units per year, generating about 50 million tonnes of waste annually [1]. This will continue to grow due to the increasing demand, population growth, and upgrades in new technologies with newly developed vehicles. Since ELVs are composed of 70% metallic parts, they are recycled as a valuable secondary source. However, the remaining components, including the automotive shredder residue (ASR), constitute 30–35% of the vehicle after the metal recovery of end-of-life vehicles (ELV), and they are polymeric. ASR is a complex mixture of several types of plastics, rubbers, foams, glasses, and textiles. A wide range of polymers used in automotive manufacturing makes the recycling of ASR difficult and inefficient [2,3]. Due to the complex mixture of variety of compounds in the ASR, the waste ends up in landfills [4]. It is estimated that between 150,000 and 200,000 tonnes of ASR are generated per annum in Victoria, Australia, alone [5]. Currently, most of the ASR is landfilled, and a small fraction is incinerated, resulting in the waste of valuable resources and severe environmental problems. The leaching of ASR landfilled contaminants releases dissolved organic compounds (DOCs), polycyclic aromatic hydrocarbons (PAHs), volatile organic compounds (VOCs), and heavy metals (Cd, Cr, Pb, and Zn) [6,7,8,9,10] into the environment. Therefore, it is imperative to investigate alternative uses of ASR. Alternative uses of ASR may include their utilization in energy plants, cement kilns, and metallurgical processes. Since the mixture is polymeric in the nature of the ASR, it can potentially be used as a source for liquid fuel generation [9,11,12] via a thermochemical process such as pyrolysis.

Pyrolysis and gasification allow the mixing of heterogeneous waste streams in a commercial process to produce valuable products such as liquid fuels, fuel gases, and chemicals. Pyrolysis of ASR produces three different fractions: (1) a solid residue (char), mainly consisting of inorganic material and unreacted carbon; (2) a liquid fraction, which consists of light and heavy condensable organic compounds; and (3) gases (H_2_, CO, CH_4_, CO_2_, C_2_H_2_, C_2_H_4_, and C_2_H_6_) produced during the decomposition process [10] The high calorific value of the plastic fraction of ASRs enables energy recovery [9,13,14,15]. In this aspect, it is necessary to develop a fundamental understanding of the thermal decomposition of ASR during pyrolysis and the associated individual functional groups and molecular structures. Furthermore, there is only limited literature on the industrial application of ASR pyrolysis products and treatment of ASR; hence, a thorough research is needed to understand the application of ASR pyrolysis products.

Techniques employed to study the ASR pyrolysis process and the devolatilisation mechanism include thermogravimetric analysis using small-scale reactors [16,17,18]. Operating pressure is a less significant factor for devolatilisation than particle size, temperature, and heating rate because mass and heat transfers have a greater dependence on particle size and temperature. There is a need of published literature on the systematic evaluation of evolved functional groups during the pyrolysis of ASR polymers and rubbers. Fourier transformation infrared (FTIR) spectroscopy is commonly used to characterise the thermal decomposition products as a temperature function [16,17,18,19,20]. Synchrotron FTIR can determine the in-situ evolution of functional groups during the pyrolysis of the polymeric ASR [21]. The effect of the heating rate on devolatilisation is another essential factor that needs to be investigated adequately.

The activation energy (E), pre-exponential factor, and reaction order can be estimated by several isothermal and non-isothermal methods (e.g., Ozawa, Coats-Redfern, Kissinger, and integral methods) for the kinetic characterisation of coal, biomass, and pyrolysis of polymers [19,20,21]. The decomposition kinetics and the reaction mechanism of ASR are complex with reaction orders not equal to one (*n* ≠ 1) [21]. The present study estimates the multi-stage heterogeneous kinetics of ASR pyrolysis and its products using the kinetic parameters estimated from the linearised form of mass loss curve for various stages of pyrolysis. 

In light of the aforementioned facts and the associated great industrial and environmental relevance, the following characteristics of the pyrolysis of three different components of a given ASR (i.e., hard black plastic/HBP, hard grey plastic/HGP, hard white plastic/HWP, and soft black rubber/SBR) were studied:

(a) Morphology and chemical characteristics of ASR and its components.
(b) Thermal analyses of ASR components for understanding the degradation kinetics and identification of the critical temperatures or temperature ranges of ASR pyrolysis. This includes synchrotron-based IR spectroscopy to examine the evolution of surface functional groups and thermogravimetric-IR to identify the gases evolved at different temperatures and heating rates. The resulting data were compared with those in the literature to characterise the active functional groups and evolved gases. For the first time, this study provides the information of surface functional groups evolved in situ during ASR pyrolysis using synchrotron IR.
(c) To obtain the kinetic parameters using multistage model.

## 2. Materials and Methods

### 2.1. Introduction

**Figure 1:**
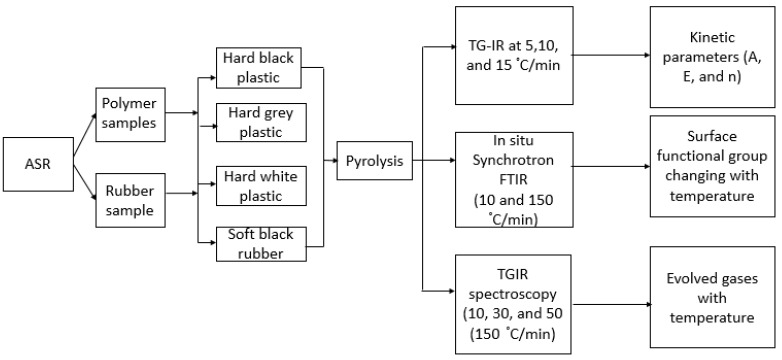
Experimental flow diagram.

ASR, as obtained from an Australian steel company, was used in this study. Polymeric components were separated and shredded in the range of 2–5 mm particle size and ground into powdered form and dried at hot air around 45 °C. The composition of the polymeric components ASR is presented in Table 1, and the entire experimental scheme is shown in Figure 1.

### 2.2. Characterization of Feedstock

**Figure 2:**
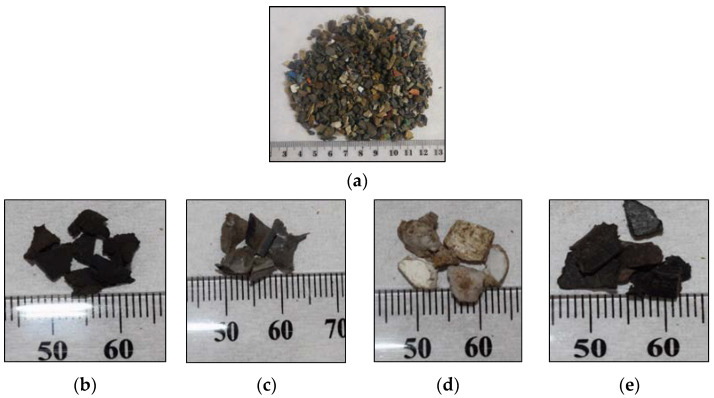
Visual appearance of (**a**) ASR raw material and its components segregated as: (**b**) hard black plastic (HBP), (**c**) hard grey plastic (HGR), (**d**) hard white plastic (HWP), and (**e**) soft black rubber (SBR).

The polymeric component of ASR was categorised into four main polymer types based on colour and texture, hard black plastic (HBP), hard grey plastic (HGP), hard white plastic (HWP), and soft black rubber (SBR), as shown in Figure 2. The composition of ASR is presented in Table 1.

#### 2.2.1. Scanning Electron Microscopy (SEM) and Energy Dispersive X-ray Spectroscopy (EDX)

Morphology and composition of ASR components (shown in Figure 2) were characterised using a scanning electron microscope (make/model: Benchtop Phenom XL, Selb, Germany). Samples were pre-dried in an oven at 45 °C before analysis. It was noted that EDX had a detection limit of ~1 wt%.

#### 2.2.2. Thermogravimetric Analysis (TGA) and Elemental Analysis

Thermogravimetric analysis was carried out using a thermal analyser (Model STA 449 F3 Jupiter, NETZSCH-Geratebau GmbH, Selb, Germany) to monitor the weight loss at different temperatures during pyrolysis. Prior the analysis, individual samples were ground to a 20–45 µm micron mesh size. Samples (15 ± 0.5 mg) were heated in an alumina crucible at the heating rates of 5, 10, and 15 °C/min. Heating was carried out in an inert atmosphere of high pure nitrogen (99.99%) at a flow rate of 100 mL/min. The sample temperature was ramped from ambient to 550 °C. The TG set-up employed an S-type thermocouple that had an accuracy of ±1.5 °C. The TG curves in the experiments were corrected by subtracting the baselines obtained in blank experiments. The composition and proximate analysis of components in ASR are presented in Table 1. The elemental components and gross calorific value of each component of ASR were calculated using the ultimate analyser (Model 2400, Perkin-Elmer, Shelton, CT, USA). The samples were combusted at 975 °C to determine the carbon, hydrogen, nitrogen, and sulphur contents. The analyser detected gases like CO_2_, N_2_, H_2_O, and SO_2_ by thermal conductivity. The heating characteristics of the material were determined by the CHNS and O percentages obtained from the CHNS unit. This is a theoretical estimate of the energy released as the material combusts in the oxygen atmosphere.

#### 2.2.3. Simultaneous Thermogravimetric and Gas-phase Infrared Spectroscopy (TG-IR)

Pyrolysis characteristics of the four components of the ASR were studied using a thermogravimetric analyser (Perkin Elmer STA 800) coupled with a Perkin Elmer Frontier spectrometer that was fitted with a detection cell. The TGA and FTIR were coupled with a heating Teflon lined tube at 270 °C by a variable transformer to minimise the condensation of the volatiles during transit as well as within the gas cell. A constant flow of 60 mL/min was maintained in the transfer line using a vacuum pump in order to facilitate the continued flow of gases though the transfer line. About 10 ± 0.5 mg of the sample was used for the weight loss experiments during the pyrolysis of the ASR components.

#### 2.2.4. In Situ Synchrotron FT-IR Spectroscopy

In situ infrared spectroscopy was conducted using the specific IRM beamline at an Australian synchrotron. The IRM beamline consisted of a Bruker V80v Fourier transform infrared (FTIR), spectrometer, and a Hyperion 2000 IR microscope, which offered a high signal-to-noise ratio at a diffraction-limited spatial resolution between 3 µm and 8 µm. The Bruker OPUS 7.2 software enabled automated multipoint selection and sample stage position. The measurements were taken using a high-sensitivity liquid-nitrogen-cooled mercury cadmium telluride (MCT) detector, optimized for the data collection at the wave number range of 4000–900 cm^−1^. The sample was placed in a Linkam transmission stage with a BaF_2_ window was capable of heating at high heating rates (e.g., 150 °C/min) under an inert atmosphere. After purging the sample chamber at 30 °C for 10 min with a flowing nitrogen stream, the samples were heated at 10 °C/min and 150 °C/min up to 550 °C in ultra-high pure nitrogen. Each IR spectrum was an aggregate of 126 scans on the individual sample particles. Sample spectra were corrected for the background. After the analysis at 550 °C, samples were cooled down to ambient temperature at a rate of 40 °C/min. For each sample, analyses were carried out on three different spots.

#### 2.2.5. X-ray Fluorescence (XRF) Analysis

The major inorganic ASR components were analysed using X-ray fluorescence spectroscopy (XRD-EDX-720; Shimadzu Corp, Kyoto, Japan), with a voltage of 15–50 kV in air. This technique offers a qualitative multi-element detection in the range from ^6^C/^11^Na to ^92^U. The mechanically mixed samples were pelletized into tablets for calibration or analysis, and triplicate runs were carried out.

### 2.3. Kinetic Analysis

Kinetic analyses were performed by the established methods. The activation energy of ASR components was calculated using the Flynn-Wall-Ozawa (FWO) and Kissinger-Akahira- Sunose (KAS) methods. The kinetic model employed is as follows.

The kinetic equation can be generally shown as follows:

(1) $$\frac{d\alpha }{dt}=k\left(T\right)f\left(\alpha \right)$$

where α is the conversion degree, t is the time, *T* is the absolute temperature, k(*T*) is the temperature-dependent rate constant, and f(α) is the temperature-independent function of conversion. The Arrhenius equation is expressed using the pre-exponential factor (A), activation energy (E), universal gas constant (R), 8.314 J/mol·K, and reaction temperature (T) and can be written as:

(2) $$k=Aexp\left(-\frac{E}{RT}\right)$$

The function f(α) in Equation (1) can be expressed as:

(3) $$f\left(\alpha \right)={\left(1-\alpha \right)}^{n}$$

where n is the reaction order. The sample conversion (α) is expressed as:

(4) $$\alpha =\frac{m_{i}-m_{t}}{m_{i}-m_{\infty }}$$

where $m_{i}$ is the initial mass of the sample, $m_{t}$ is the mass at time t or temperature (T), and $m_{\infty }$ is the final mass after the reaction. The combination of Equations (2) and (3) gives:

(5) $$\frac{d\alpha }{dt}=A\exp(-\frac{E}{RT})\;{\left(1-\alpha \right)}^{n}$$

(6) $$\beta =\frac{dT}{dt}$$

where β is the heating rate.

Equation (6) can be substituted into Equation (5) and becomes:

(7) $$\frac{d\alpha }{{\left(1-\alpha \right)}^{n}}=\frac{A}{\beta }\exp(-\frac{E}{RT})dT$$

The integrated form of Equation (7) can be expressed as:

(8) $$G\left(\alpha \right)=\int_{0}^{\alpha }\frac{d\alpha }{f\left(\alpha \right)}=\frac{A}{\beta }\int_{T_{0}}^{T}\exp(-\frac{E}{RT})dT$$

The two most commonly used methods for determining the kinetic parameters of pyrolysis are the Flynn-Wall-Ozawa (FWO) and Kissinger-Akahira-Sunose (KAS) methods. Both of these methods can be used to determine the activation energy *E* of the sample. Flynn-Wall-Ozawa (FWO) is a simple method, and it was developed to determine the activation energies directly from the data obtained using several different heating rates. The equation used can be noted as,

(9) $$\ln\left(\beta \right)=ln\frac{0.0084AE}{RG\left(\alpha \right)}-1.0516\;\frac{E}{RT}$$

The Kissinger-Akahira-Sunose (KAS) method is based on the following equation:

(10) $$\ln\left(\frac{\beta }{T^{2}}\right)=ln\left[\frac{AR}{EG\left(\alpha \right)}\right]-\frac{E}{RT}$$

For α = constant, the plots between ln(β) vs. 1/*T* and $\ln\left(\beta /T^{2}\right)$ vs. 1/*T* yields straight lines whose slope allows for the evaluation of the apparent activation energy. *R* in Equations (9) and (10) is a universal gas constant which is equal to 8.314 J/mol·K.

## 3. Results and Discussions

### 3.1. Proximate and Ultimate Analysis

The calorific values, proximate and ultimate analysis of ASR components are listed in Table 1. The main plastics found in ASR are polypropylene (PP), polyesters (PET/PBT), and polycarbonates (PCs), as shown in Table 2. The ASR components exhibited different properties and calorific values.

HGP had a higher calorific value than all the other ASR components, mainly because of its high volatile content, and was expected to have less char remaining when pyrolyzed. HGP had higher carbon and hydrogen contents than the other components. The SBR had the higher moisture content compared to other ASR components. The overall calorific value of all ASR components was in the range from 17.57 to 32.60 MJ/kg, which implies that recycled ASR may be a promising fuel source.

### 3.2. Morphology and Broad Chemical Composition of Different ASR Components and Their Pyrolysis Products

**Figure 3:**
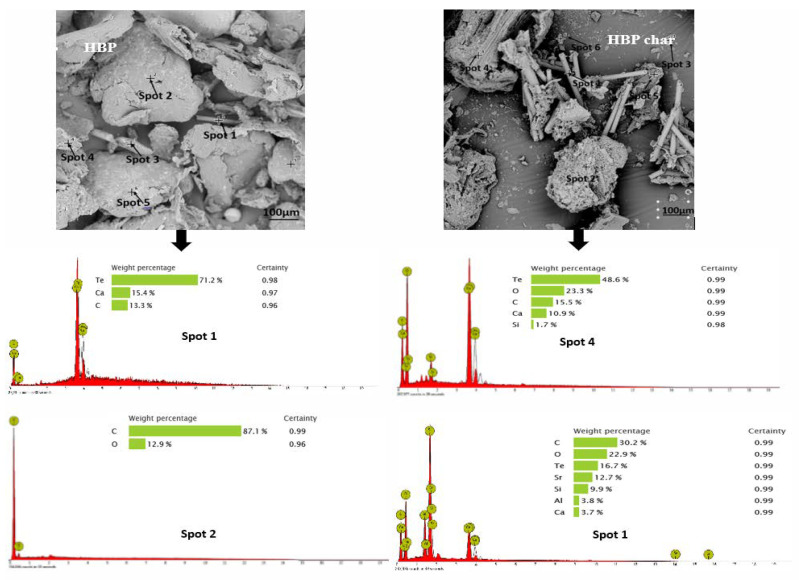

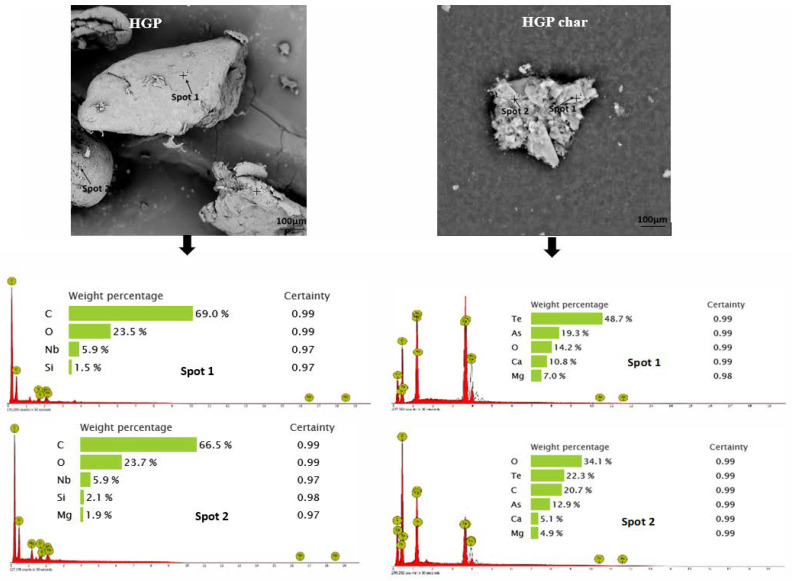

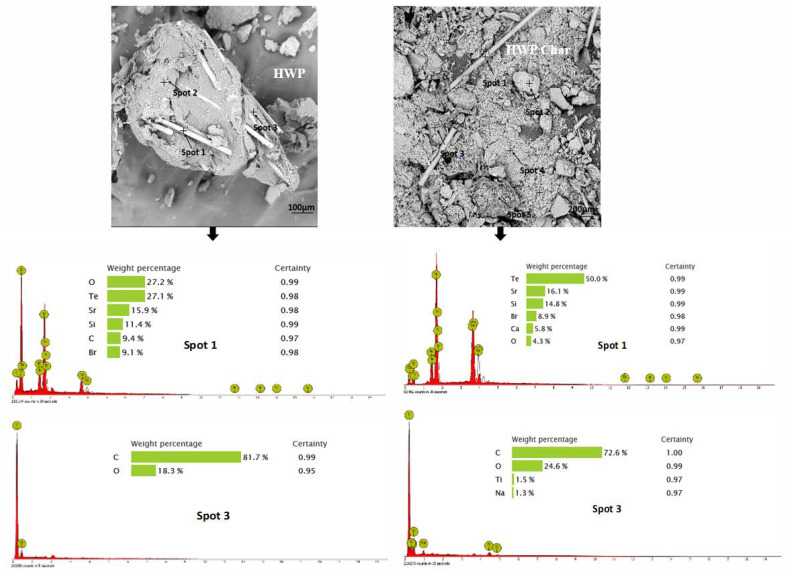

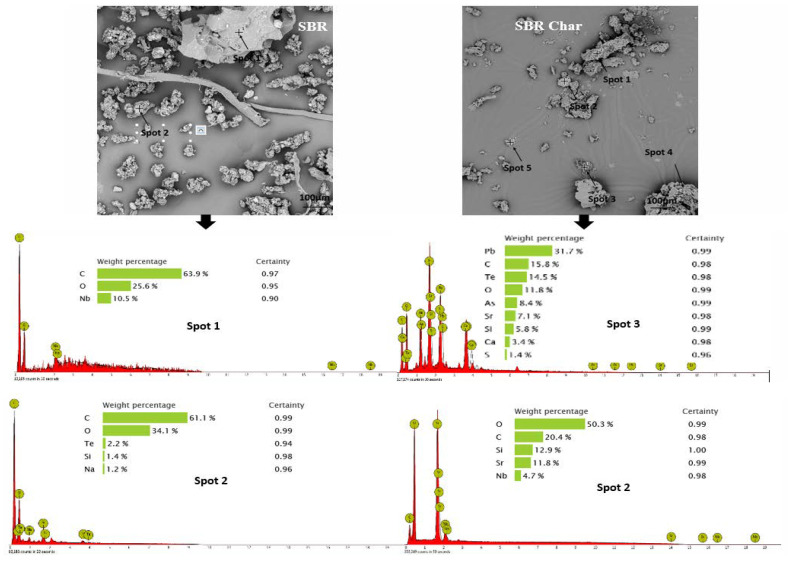
SEM and EDX results for the raw ASRs and their chars (Note: elements < 1% are below the detection limit of EDX analysis).

The ASR components (Figure 2b–e) and their chars examined using SEM and the EDX at different spots, shown in Figure 3, indicate variations in physical and chemical nature. There were no obvious observations of particle swelling, fragmentation, or agglomeration in the chars. The porous surface indicates gases being evolved. The rod-shaped structures were identified to be the oxides of Te, Sr, Si, As, Dy, Ti, and Nb. As the decomposition temperatures of these oxides are much higher than the temperature of pyrolysis employed in the study (i.e., 550 °C), the oxides were preserved in the ash of the chars retaining their rod shapes. The recovery of Te, Sr, Dy, Ti, and Nb from ASRs can add to the commercial value, i.e., over and above the production of liquid fuel.

### 3.3. TG-IR and In Situ Synchrotron FTIR of Different ASR Components

**Figure 4:**
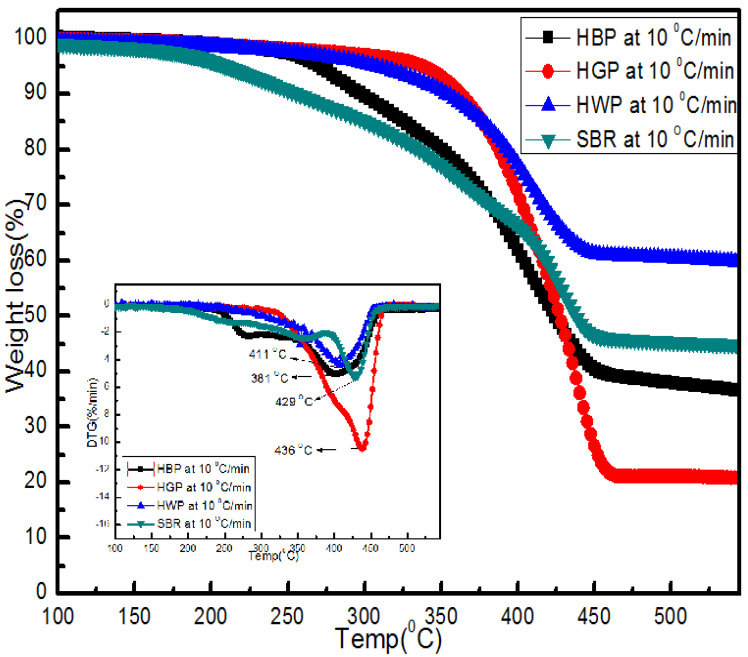
TG and DTG plots of different ASR component types (HBP, HGP, HWP, and SBR) during their heating to 550 °C at the heating rate of 10 °C/min.

The TG and DTG plots of the ASR components during heating to 550 °C at 10 °C/min are presented in Figure 4. The maximum degradation temperatures of the ASR components varied between 378 and 480 °C. The degradation temperatures of the ASR components can vary from the reported values for the virgin plastics (Table 3) due to the contaminants present in the ASRs.

#### 3.3.1. Hard Black Plastic (HBP)

As shown in Figure 4, HBP had significant weight loss in the temperature range of 211–470 °C, with the maximum weight loss occurring at ~399 °C. The total weight loss corresponded to the volatile matter as determined in the proximate analysis (Table 1). HBP pyrolysis occurred in two stages. The first stage (211–290 °C) weight loss accounted for 21% of the total weight loss, and the second stage accounted for 41% of the total weight loss. The mass of gases produced during stage 1 was much lower compared to stage 2. At the maximum degradation temperature, 290 °C of the first stage, the degradation of oxygen-containing functional groups, (e.g., carbonyl, hydroxyl, or carboxyl groups) and impurities (e.g., additives, stabilizers) present in the ASRs occurred. The main gases that evolved around 300 °C were CO, CO_2_, and negligible amounts of CH_4_. The oxygen-containing functional groups in ASRs, as impurities, can decompose into carbon dioxide, carbon monoxide, and water at elevated temperatures via demethoxylation, demethylation, and decarboxylation reactions [30].

At the maximum degradation temperature of 399 °C, during the second stage, the weight loss could be due to the thermal degradation of the polymer chain, such as scission reactions. The breakage of C-C and C-H bonds in this temperature regime can lead to the formation of alkanes, alkenes, aromatics, and CO_2_ [25,31]. The main gases formed around 400 °C were CO, CO_2_, CH_4_, and negligible amounts of C_2_H_4_ and C_2_H_6_. The gases formed around 500 °C were negligible amounts of H_2_ [10]. The residual weight of the HBP at the end of the two stages at 550 °C was ~37% of the original weight, which was attributed to ash and fixed carbon (Table 1).

**Figure 5:**
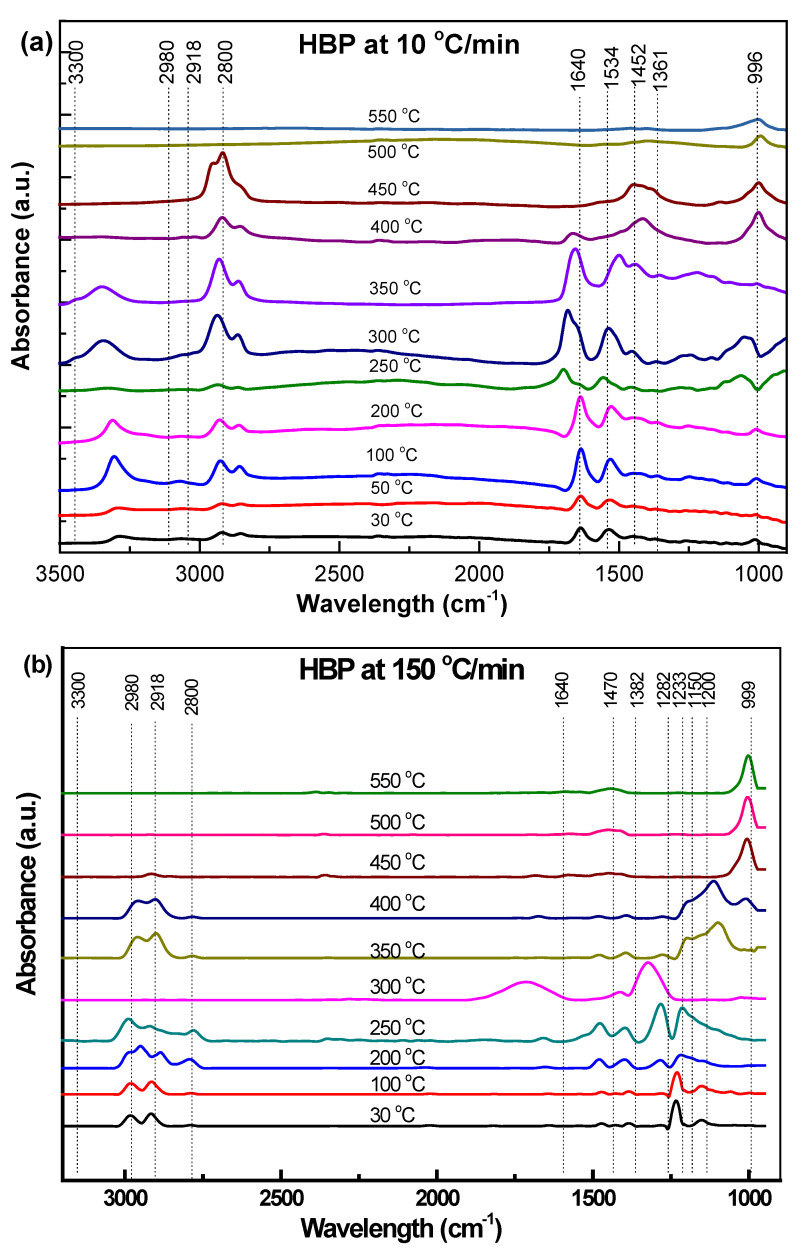
Effect of temperature on the functional group evolution of HBP: (**a**) 10 °C/min and (**b**) 150 °C/min.

**Figure 6:**
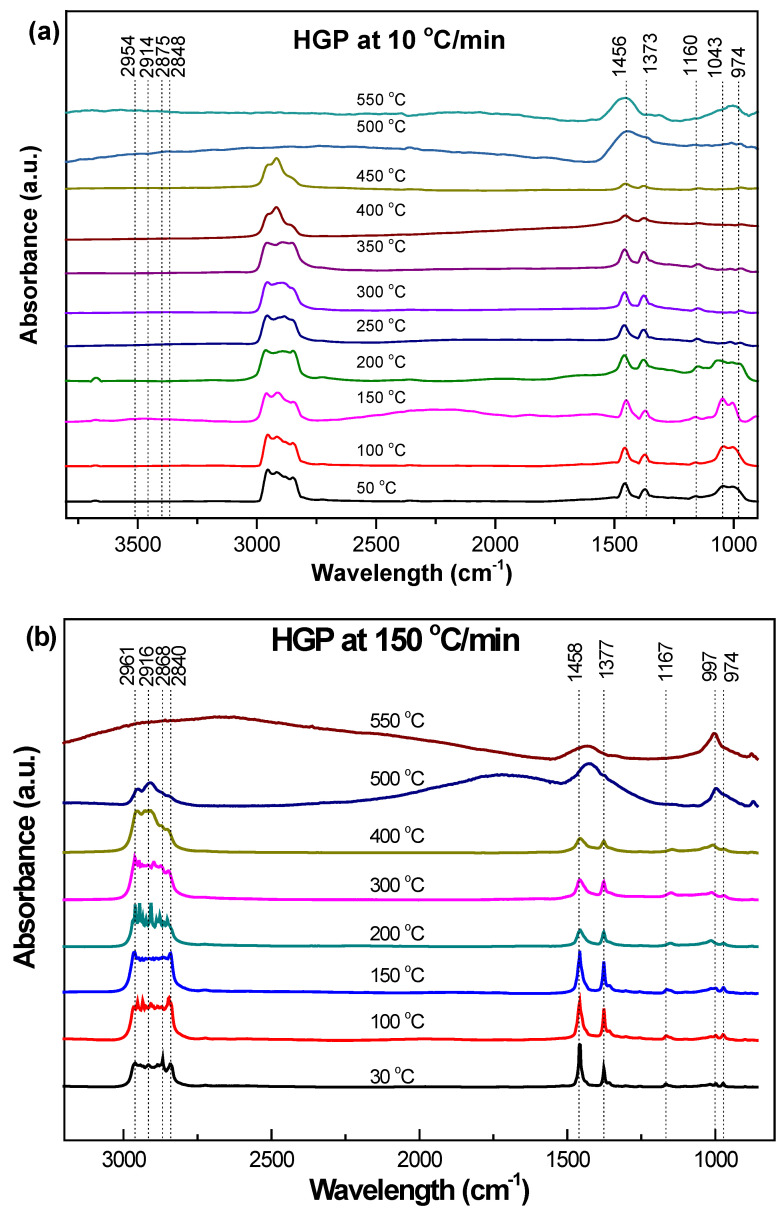
Effect of temperature on the functional group evolution of HGP: (**a**) 10 °C/min and (**b**) 150 °C/min.

**Figure 7:**
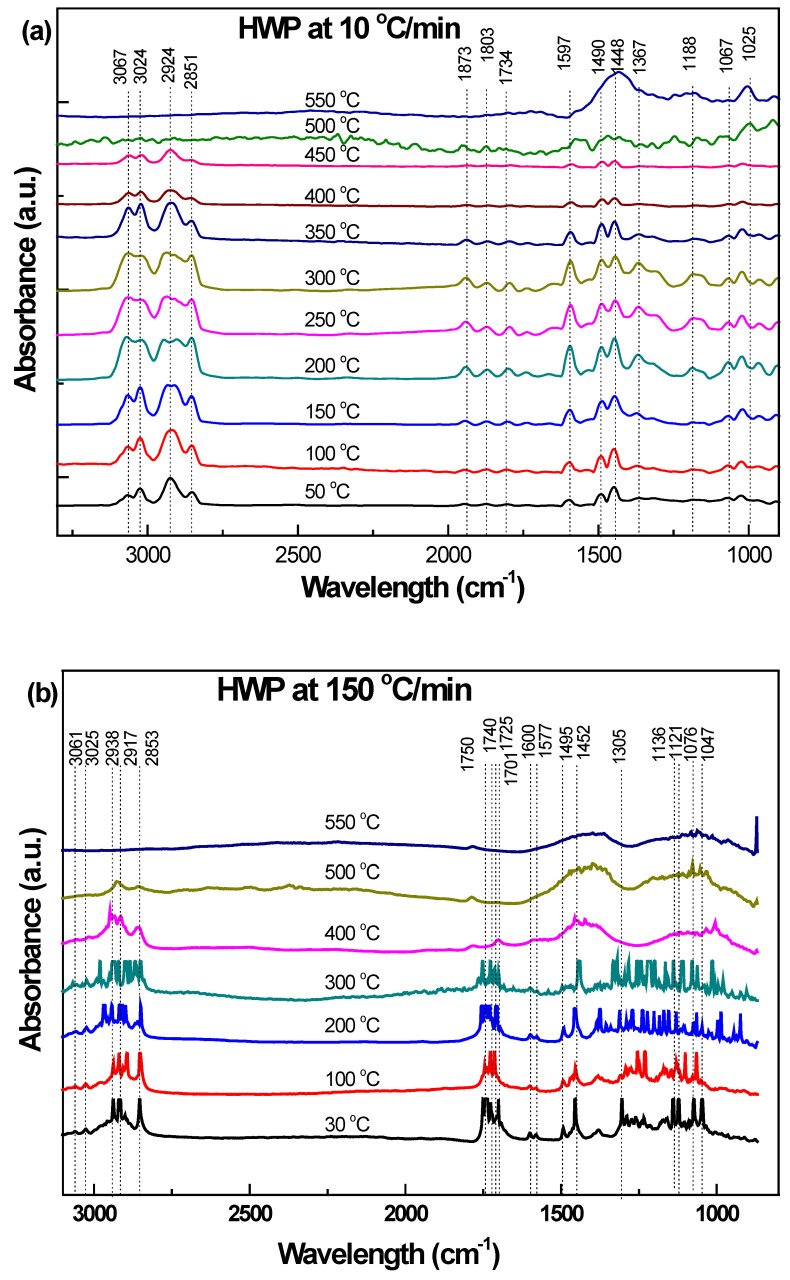
Effect of temperature on the functional group evolution of HWP: (**a**) 10 °C/min and (**b**) 150 °C/min.

**Figure 8:**
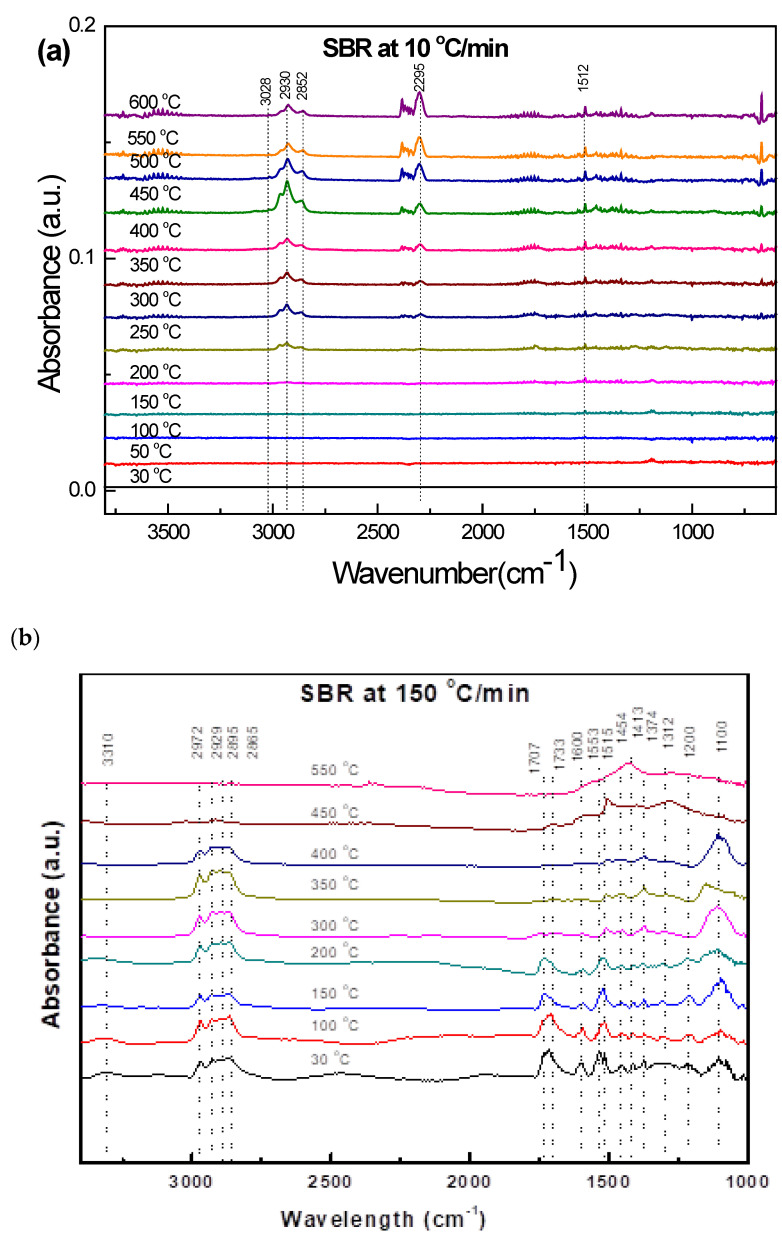
Effect of temperature on the functional group evolution of SBR (**a**) 10 °C/min and (**b**) 150 °C/min.

Figure 5a, Figure 6a and Figure 7a and Figure 5b, Figure 6b, Figure 7b and Figure 8b show the in-situ synchrotron FTIR spectra for HBP, HGP, HWP, and SBR at different stages of heating at 10 °C/min and at 150 °C/min, respectively. The characteristic IR peaks for the different ASR component types and the actual peaks detected in the synchrotron FTIR spectra during heating to 550 °C at 10 °C/min and 150 °C/min are presented in Table 4, Table 5, Table 6 and Table 7.

Figure 5a,b presents the in-situ synchrotron FTIR spectra of HBP, generated during heating from ambient to 550 °C at different 10 °C/min and 150 °C/min. The characteristic IR peaks for the typical functional groups in HBP and the peaks actually detected in the synchrotron FTIR spectra during heating to 550 °C, at 10 °C/min and 150 °C/min, are presented in Table 4.

The spectra clearly indicate the evolution of new functional groups with the increase in temperature. However, some differences were observed between the HBP spectra obtained at 10 °C/min and 150 °C/min, which may be attributed to the different responses of the functional groups due to the change in the heating rate [37]. Also, there were some shifts in the peaks from 2980 cm^−1^, 2918 cm^−1^, and 2800 cm^−1^ at 150 °C/min (to 2958 cm^−1^, 2926 cm^−1^, and 2855 cm^−1^ 10 °C/min, respectively), which is generally the result of the change in adjacent environment and molecular interaction, which, in turn, is likely to be influenced when the heating rates are different as in the present case. The shift in the peak is also attributed to the release of functional groups at different heating rates. Figure 5 suggests that the 150 °C/min, there were less functional groups beyond 400 °C, i.e., the low molecular weight of the sample caused the shift in the peaks toward higher wave numbers compared to 10 °C/min where the functional groups were still available beyond 450 °C.

The peak intensities of all the functional groups (including the new/evolving ones) increased with the pyrolysis temperature, both at 10 °C/min and 150 °C/min (Figure 5a,b). However, such increases in intensity with the pyrolysis temperature in stage 1 (211–290 °C) were not as prominent at the lower heating rate (10 °C/min). In stage 2 (355–470 °C), the intensity of most functional groups began to decrease and disappear during heating above 350 °C and 400 °C, respectively. This suggests the evolution of small aliphatic and weakly bonded non-covalent groups, as well as the evolution of C-C and C-O and C-N (the latter gradually continued until 500 °C). The results obtained from TG are consistent with those in the in situ FTIR spectra, where the maximum degradation, i.e., loss of functional groups, was seen to occur around ~400 °C. At 450 °C, methylene CH_2_ stretching and deformation vibrations at 2980–2918 cm^−1^ and 1452–1361 cm^−1^ were, respectively, visible; however, amide C=O stretching at 1640 cm^−1^ and amide II N-H bending and C-N stretching at 1534 cm^−1^ completely disappeared, which can be related to the release of CO, CO_2_, CH_4_, C_2_H_4_, C_2_H_6_, and NO_2_. All functional groups reached a plateau, except C-stretching and bending vibrations at 996 cm^−1^ at 500 °C. At low temperatures (300 °C), light-molecular-weight compounds were produced. 

At the higher heating rate (150 °C/min), most peaks were in the same wave number range (with respect to their corresponding positions in the spectrum at 10 °C/min) except for the one corresponding to the C-C stretching and bending vibration at 999 cm^−1^ (at 400–500 °C). Higher heating rates and high sample temperatures are known to shift characteristic peaks to higher wave numbers [21]. The amide C=O stretching at 1640 cm-^−1^ bond completely disappeared at 400 °C. The peak at ~999 cm^−1^ became prominent at 400 °C at 10 °C/min and beyond 450 °C for 150 °C/min heating rates, which could have been due to the presence of chain terminating unsaturated (-CH=CH_2_) groups that formed because of the chain scission.

#### 3.3.2. Hard Grey Plastic (HGP)

Figure 4 shows that the pyrolysis of HGP occurred in a single stage in contrast to the two-stage decomposition of HBP. The maximum degradation of HGP occurred at a relatively higher temperature (436 °C) compared to HBP (399 °C). The total weight loss of 82% HGP (which was considerably greater than that for HBP, i.e., 62%) was due to the devolatisation from the scission reaction (that led to the formation of mainly oligomers of alkanes and alkenes during the breakage of C-C at elevated temperatures [38]). With the total weight loss (82%), the fixed carbon and ash left of HGP at the end of the degradation stage at 550 °C was only 18%.

In-situ synchrotron FTIR spectra generated during heating of HGP from ambient to different temperatures up to 550 °C at 10 °C/min and 150 °C/min are presented in Figure 6a and 6b, respectively. The nature of the spectra obtained from HGP was quite similar to those for HBP. However, unlike HBP, there were no noticeable differences between the spectra for 10 °C/min and 150 °C/min. Peak shifts were also observed for HGP in the 2954–2961 cm^−1^ range, which can be attributed to the change in the adjacent environment during heating at the two heating rates. However, oxygenated carbonyl and amide II and III groups were absent in the case of HGP. Moreover, HGP presented four superimposed aliphatic absorption bands that corresponded to the stretching vibrations of methyl and methylene groups, while HBP presented only two aliphatic -CH_2_- stretching vibrations. The most prominent peaks in the in-situ synchrotron FTIR spectra for HGP are shown in Table 5. Whereas methyl and methylene groups were present in HGP, HBP showed only two aliphatic -CH_2_-stretching vibrations.

The four prominent peaks for aliphatic constituents (2961–2848 cm^−1^) were reduced to three at 400 °C. This seems to have a correlation with the substantial weight loss seen at ~436 °C in the TG/DTG plots (at 10 °C/min), which is attributed to the loss of the volatile matter (as seen in the spectra at ≥400 °C).

Similar to the IR spectra at 10 °C/min for HBP, the aliphatic C-H stretch at 2916–2961 cm^−1^ completely vanished at 500 °C, as well as the peaks corresponding to C-H and RCH=CH_2_ bending (1373–1459 cm^−1^ and 1000–974 cm^−1^, respectively) became fewer in number. Again, unlike the IR spectra at 10 °C/min for HBP, there was a lag in the evolution of functional groups at the higher heating rate (150 °C/min). For both high and low heating rates, the extent of evolution and remaining functional groups at 550 °C were similar. This is important information for optimizing the conditions for the pyrolysis of plastics. These spectra resemble the reference spectrum of polypropylene [38].

#### 3.3.3. Hard White Plastic (HWP)

Figure 4 indicates a total weight loss of 48.6% in a single step for HWP, and the least among all the ASR components which corresponds to the volatile matter present in the sample (Table 1). The maximum degradation temperature was 411 °C, which was in between that of HBP at 399 °C and HGP at 436 °C. The total weight left at the end of the decomposition, 50.58% (which was relatively higher than that of HBP and HGP), corresponded to 28.36% ash and 22.22% fixed carbon.

In situ synchrotron FTIR spectra generated during the heating of HWP from ambient temperature to 550 °C at 10 °C/min and 150 °C/min are presented in Figure 7a,b. Table 6 compares the synchrotron FTIR peaks with those reported in the literature. The spectrum for HWP was significantly different from the spectra for HBP and HGP at 10 °C/min and 150 °C/min.

Like HBP and HWP, peak shifts for HWP were observed at both the heating rates. It is suggested that a high temperature and/or a high heating rate can shift characteristic peak to a higher wave number. Scission reactions (resulting in lower mass) can shift the peak to higher wave numbers. Wave numbers of both stretching as well as bending vibrations are changed because of hydrogen bonding. Stretching bands move to lower frequencies, usually with increased intensity and band widening. The bending vibration usually shifts to higher frequencies. High temperatures decrease the extent of hydrogen bonding, thus causing peak shifts [34].

Table 6 shows the main peaks identified in the in-situ synchrotron spectra for HWP degradation. In addition to the functional groups detected in HBP and HGP, the spectra for HWP also consisted of peaks corresponding to aromatic stretching (3100–3000 cm^−1^) and bending of the monosubstituted benzene ring (2000–1700 cm^−1^).

At the lower heating rate, the evolution of functional groups was the same during heating to various temperatures until 350 °C. However, these groups started to disappear at or above 400 °C. TG/DTG curves were consistent with IR data, suggesting sudden and substantial changes above 411 °C. The intensity of aliphatic bands (3000–2800 cm^−1^) and the overtone bands (2000–1700 cm^−1^) substantially decreased at 500 °C. It was noted that the overtone bands (2000–1700 cm^−1^) that corresponded to mono-substituted benzene rings were exclusive for HWP. At 500 °C, almost all the functional groups disappeared.

At the higher heating rate (150 °C/min), strongly bonded functional groups (namely methylene C-H stretching, aromatic C=C stretching and methyl C-H bending, and ether C-O-C stretching) and aromatic C-H bending were still observed at 500 °C. This contrasts with the corresponding peaks for HBP (where most functional groups disappeared and reached a plateau at lower temperatures at the high heating rate). This is because HWP has a higher ash content (28.36%) than HBP (17%). In the industrial sense, these results suggest that HGP and HWP would require a relatively higher pyrolysis temperature (550 °C) and high heating rates for the maximum evolution of functional groups. The bands present are consistent with those for polystyrene [37].

#### 3.3.4. Soft Black Rubber (SBR)

Figure 4 shows that SBR degradation occurred in two-steps in the temperature range of 162–475 °C, with the maximum weight loss in the first stage (at 358 °C) accounting for 32% weight loss at 10 °C/min, suggesting the presence of additives and rubber [29,39,40]. This region corresponds to the reduction of oxygen-containing, carboxyl, carbonyl, and hydroxyl groups, mainly due to the C=O and O-H stretching vibration of the corresponding acids, ketones, and alcohol groups. These oxygenated compounds can decompose into carbon dioxide, carbon monoxide, and water at elevated temperatures. The second stage, for which the maximum degradation was at 429 °C, accounted for the 23% weight loss that corresponded to the main rubber decomposition. For the final weight of SBR, 45% corresponded to 23.52% ash and 20.34% fixed carbon. From the literature, the maximum decomposition temperatures reported for tyre rubber are in the temperature range of 378 °C for natural rubber (NR), 447 °C for styrene-butadiene rubber (STBR), 467 °C for butadiene rubber (BR), and 427 °C for a blend of butadiene rubber with styrene-butadiene rubber. Therefore, the first decomposition occurring below 300 °C is attributed to the additives and/or oils used in the preparation of tyres, and the second decomposition stage is attributed to the main rubber decomposition [28,29].

In situ synchrotron FTIR spectra generated during heating of SBR from ambient to different temperatures up to 550 °C are presented in Figure 8. The main functional groups identified from the in-situ synchrotron FTIR spectra for SBR are presented in Table 7. IR spectra indicated that most functional groups disappeared in rubber sample at around 450 °C at 150 °C/min. This corresponds to the decomposition of C--H stretching of aliphatic groups and C=O stretching of the oxygen-containing functional groups. The alkene and carbonyl regions (1800–1500 cm^−1^) mostly disappeared at 300 °C. At 450 °C, a fewer C=O and aliphatic C-H stretches were still observed, which became quite low or invisible at 550 °C. The oxygen-containing groups in rubber can further generate CO, CO_2_, and water vapours. When the sample was heated at 10 °C/min, the evolution of gases appeared from 250 °C with hydrocarbon scission and methane and CO_2_ releasing from the sample.

Thus, the combined TG/DTG and synchrotron IR results provide crucial information on the temperature regime, role of the heating rate, chemical bond breakdown, and the evolution of functional groups during pyrolysis, which is not only applicable for effective industrial pyrolysis of ASRs but can also be critical in understanding the mechanism of ASR pyrolysis that needs further investigation. These results can be compared with commercially available references to identify the synthetic polymer, which is correlated to polyamide present in ASR.

### 3.4. TG/DTG/DSC and Simultaneous IR Analysis of Evolved Gases

The pyrolysis of the ASR components generated volatile products. The simultaneous TG/DTG/DSC and IR analysis presented in this section made it possible to concurrently characterise the volatile matter as they evolved during the pyrolysis. Whereas the synchrotron IR spectra at different temperatures indicate the evolution of surface functional groups, the gas-phase IR allowed for the characterisation of the gases evolved. The gas-phase IR spectra presented in this section may corroborate the solid-state residues analysed by synchrotron IR. It was noted that the gas-phase IR spectra for each of the ASR component types (viz., HBP, HGP, HWP, and SBR) had distinct peaks for CO_2_ (2400 cm^−1^), CH_4_ (1306 cm^−1^, 3016 cm^−1^), and water (1500–1700 cm^−1^).

#### 3.4.1. HBP

**Figure 9:**
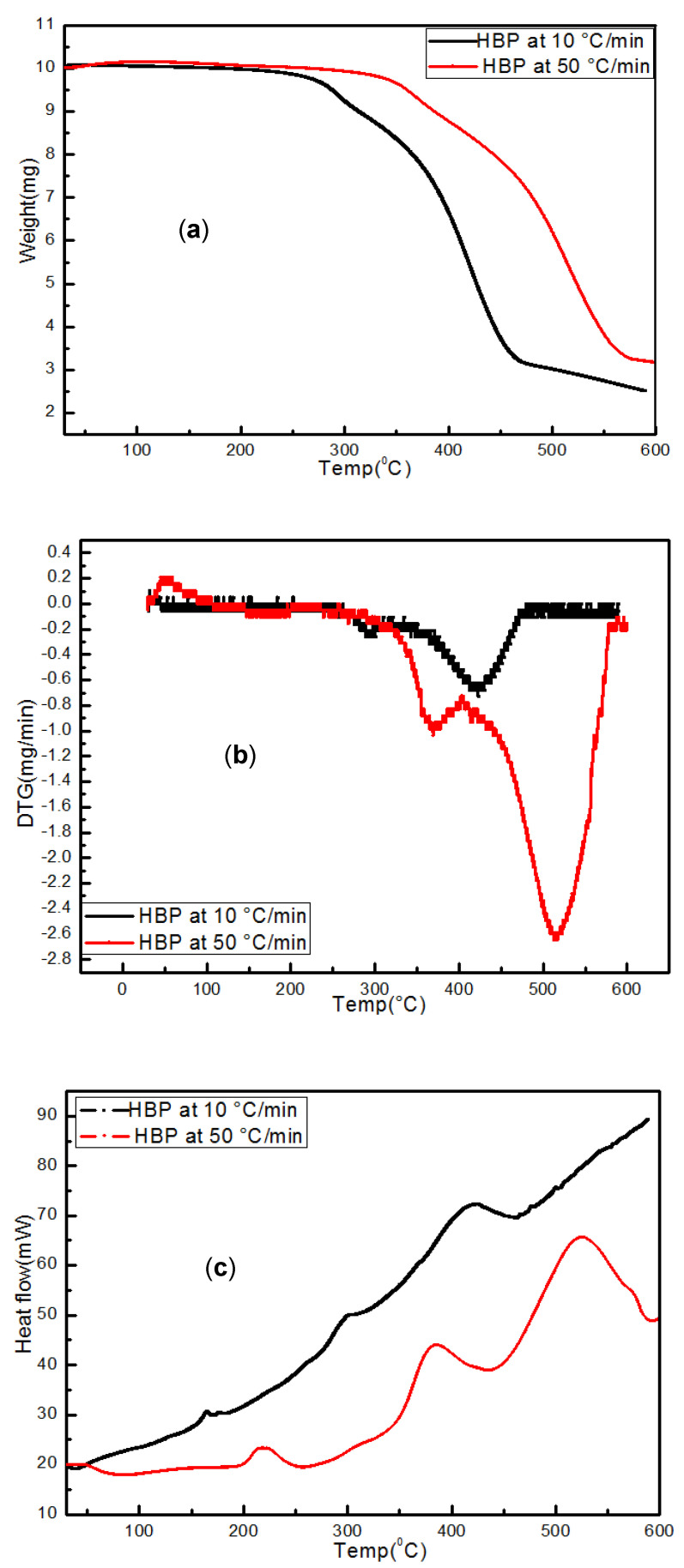

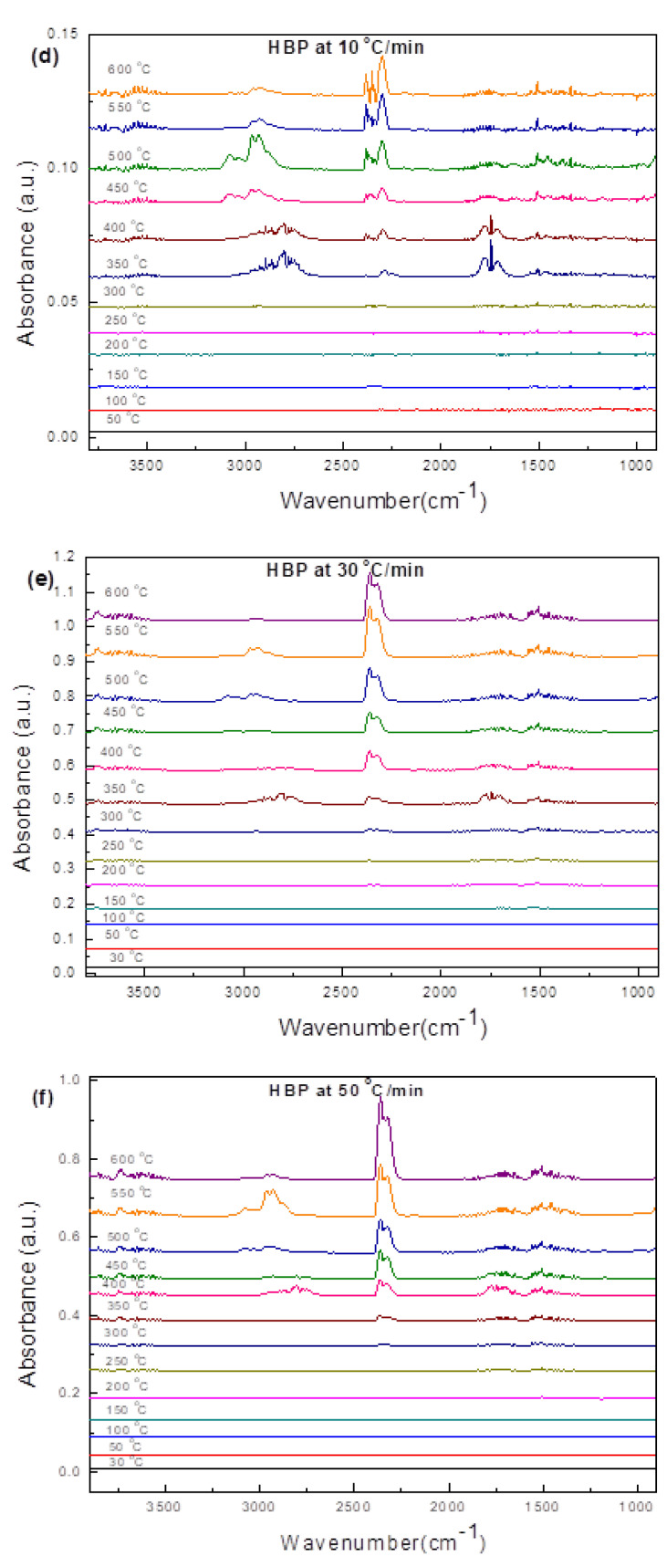
(**a**–**c**) Thermal analyses (TG/DTG/DSC) data of HBP during heating at 10 and 50 °C/min to 600 °C. (**d**–**f**) Corresponding evolved gas IR spectra of hard black plastic (HBP) during heating to different temperatures at the two rates.

TG/DTG/DSC of pyrolyzed hard black plastic (HBP) at 10 and 50 °C/min are presented in (Figure 9a–c). The weight loss over the two stages and residual char at the two heating rates was in the range of 27–35%, which is consistent with the TG/DTG data in Figure 4. The TG/DTG results indicate (Figure 4) that oxygen-containing functional groups degraded into CO_2_, CO, and H_2_O around 350 °C (i.e., the first stage) in the HBP. The gas-phase IR spectra, when heating up to 600 °C (Figure 9d,e), confirmed the peaks for CO_2_ (2355 and 2325 cm^−1^) and H_2_O (1682 and 1500 cm^−1^), as well as methane (~3020 cm^−1^) above 350 °C. The DSC curves showed peaks at 200 °C, which is typical of melting of HBP plastics.

The synchrotron IR and TG/DTG results for HBP suggest the breakage of C-C and C-H bonds at ~400 °C, forming alkanes, alkenes, aromatics, and CO_2_. The gas-phase IR spectra generated during heating up to 600 °C (Figure 9d,e) showed peaks for alkanes and aromatics above 350 °C at 10 °C/min. These alkanes (2956 cm^−1^) and aromatic (3080 cm^−1^ and 3035 cm^−1^) peaks were also observed during heating at 50 °C/min, but at higher temperatures, i.e., the transformations lagged.

#### 3.4.2. HGP

**Figure 10:**
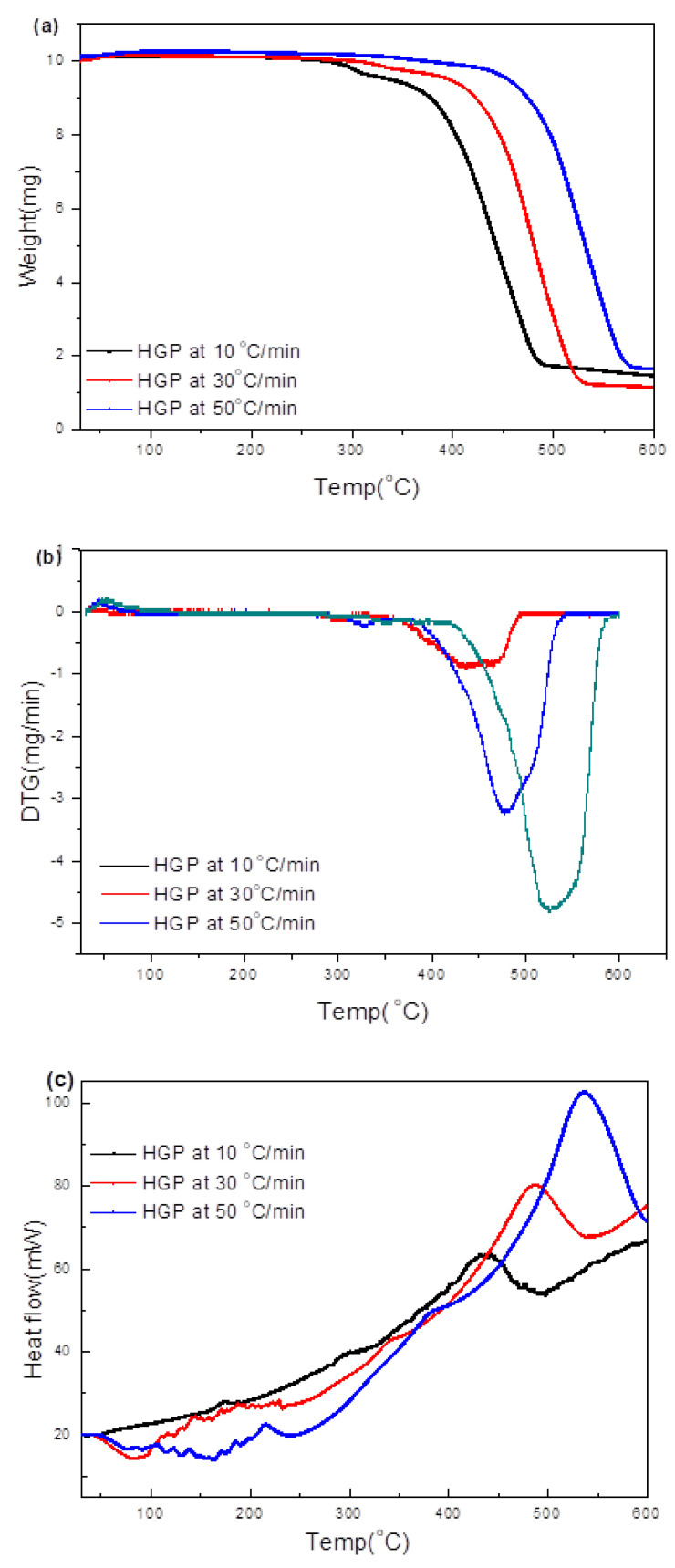

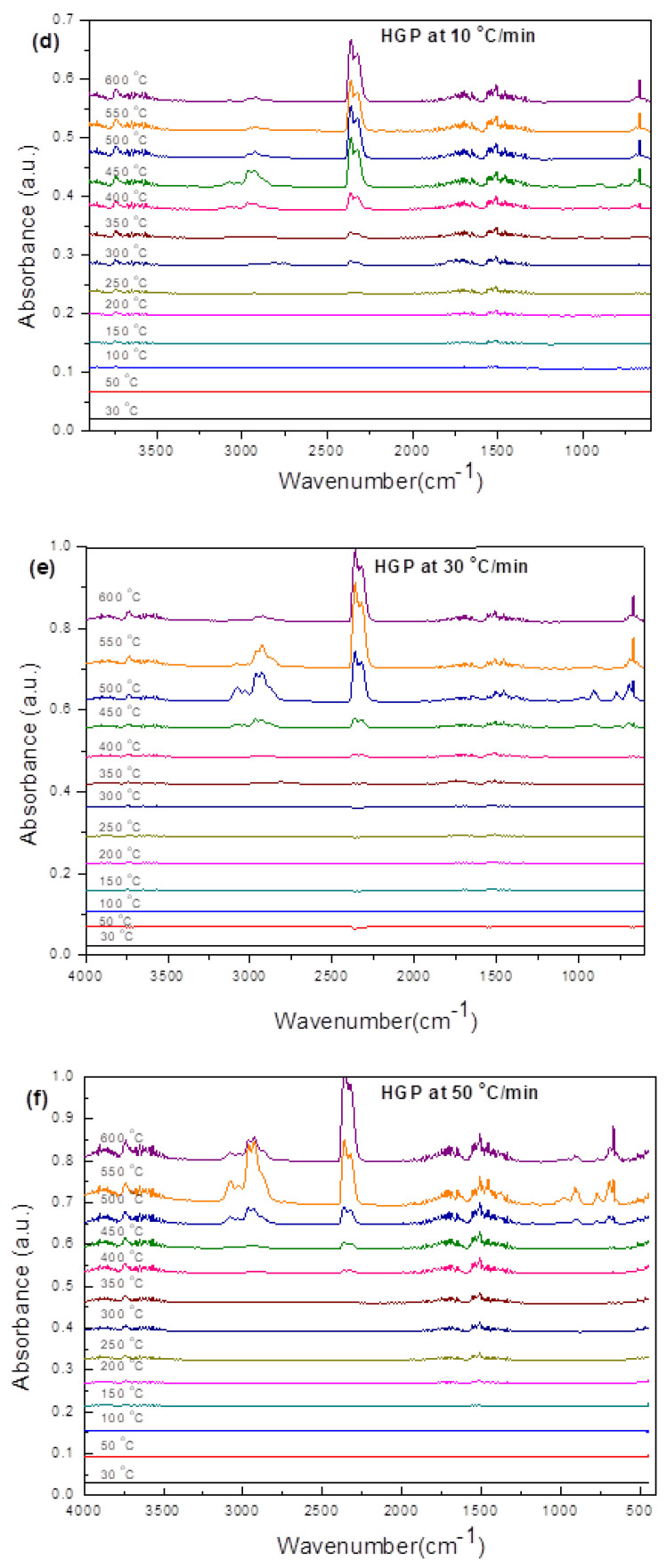
(**a**–**c**) Thermal analyses (TG/DTG/DSC) data of HGP during heating at 10, 30, and 50 °C/min to 600 °C. (**d**–**f**) Corresponding evolved gas IR spectra of hard grey plastic (HGP) during heating to different temperatures at the three rates.

Figure 10a–c shows the TG/DTG/DSC plots for pyrolysis of HGP at 10, 30, and 50 °C/min. Weights of the residue chars after the pyrolysis reaction at three different heating rates were in the range of 10–20%, which is consistent with the TG/DTG results (18%), as was the case for HBP (Figure 4). The IR along with the TG/DTG results at 10 °C/min (Figure 5) suggest the degradation of HGP to be a one-step process, with the maximum degradation at ~436 °C, which is consistent with the TG/DTG results in Figure 4. The prominent IR peaks for the gases were observed at all heating rates (i.e., 10, 30, or 50 °C/min). Peaks in Figure 10d–f correspond to: 1. methane (1306, 3020 cm^−1^) becoming prominent above 400 °C at 10 °C/min and 30 °C/min and above 450 at 50 °C/min, 2. CO_2_ and water (~2400 cm^−1^) becoming prominent above 300 °C at 10 °C/min, above 350 °C at 30 °C/min, and above 400 at 50 °C/min during heating at ≥400 °C.

#### 3.4.3. HWP

**Figure 11:**
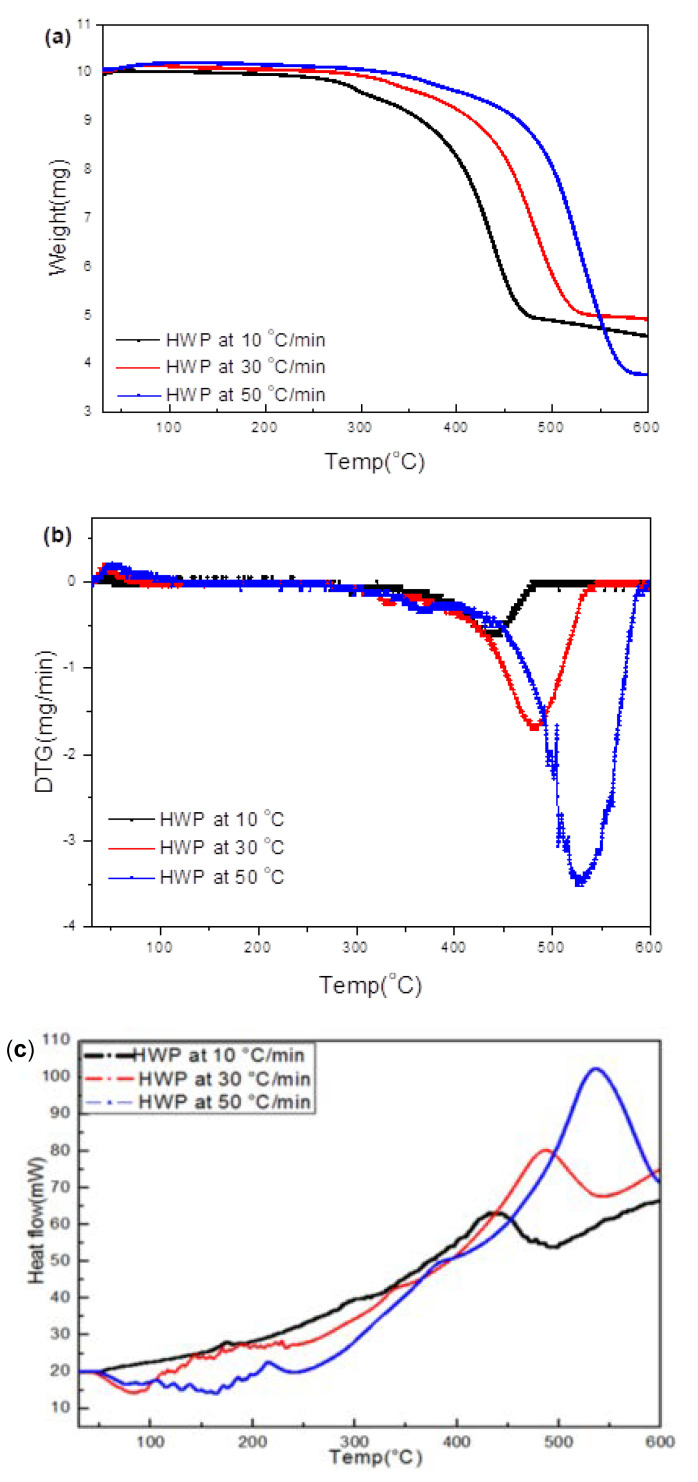

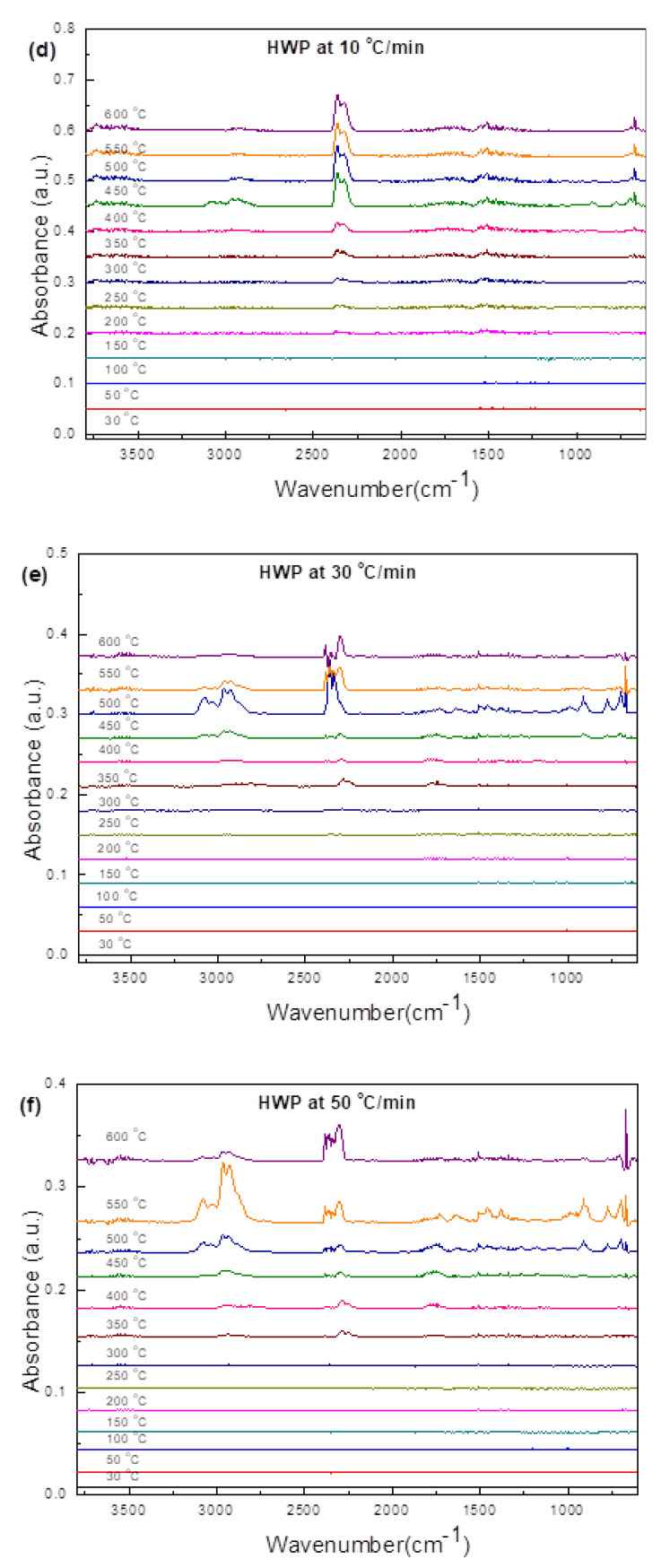
(**a**–**c**) Thermal analyses (TG/DTG/DSC) data of HWP during heating at 10, 30, and 50 °C/min to 600 °C. (**d**–**f**) Corresponding evolved gas IR spectra of hard white plastic (HWP) during heating to different temperatures at the three rates.

TG/DTG/DSC plots (Figure 11a–c) for pyrolysis of HWP at 10, 30, and 50 °C/min suggest that pyrolysis occurs in a single step, with the residual weigh in the range of 35–50%, which is consistent with the TG/DTG results (50% in Figure 4). The maximum degradation was ~425 °C at 10 °C/min, which is reasonably close to 411 °C that was detected in the TG/DTG results shown in Figure 4. The most obvious peaks in the gas-phase IR spectra at the three heating rates (10, 30, and 50 °C/min) in Figure 11d–f correspond to CH4, CO_2_, and H_2_O. CH_4_ was observed above 450 °C at all heating rates; the CO_2_ peaks were observed above 250 °C at 10 °C/min and above 350 °C at 30 and 50 °C/min.

#### 3.4.4. SBR

**Figure 12:**
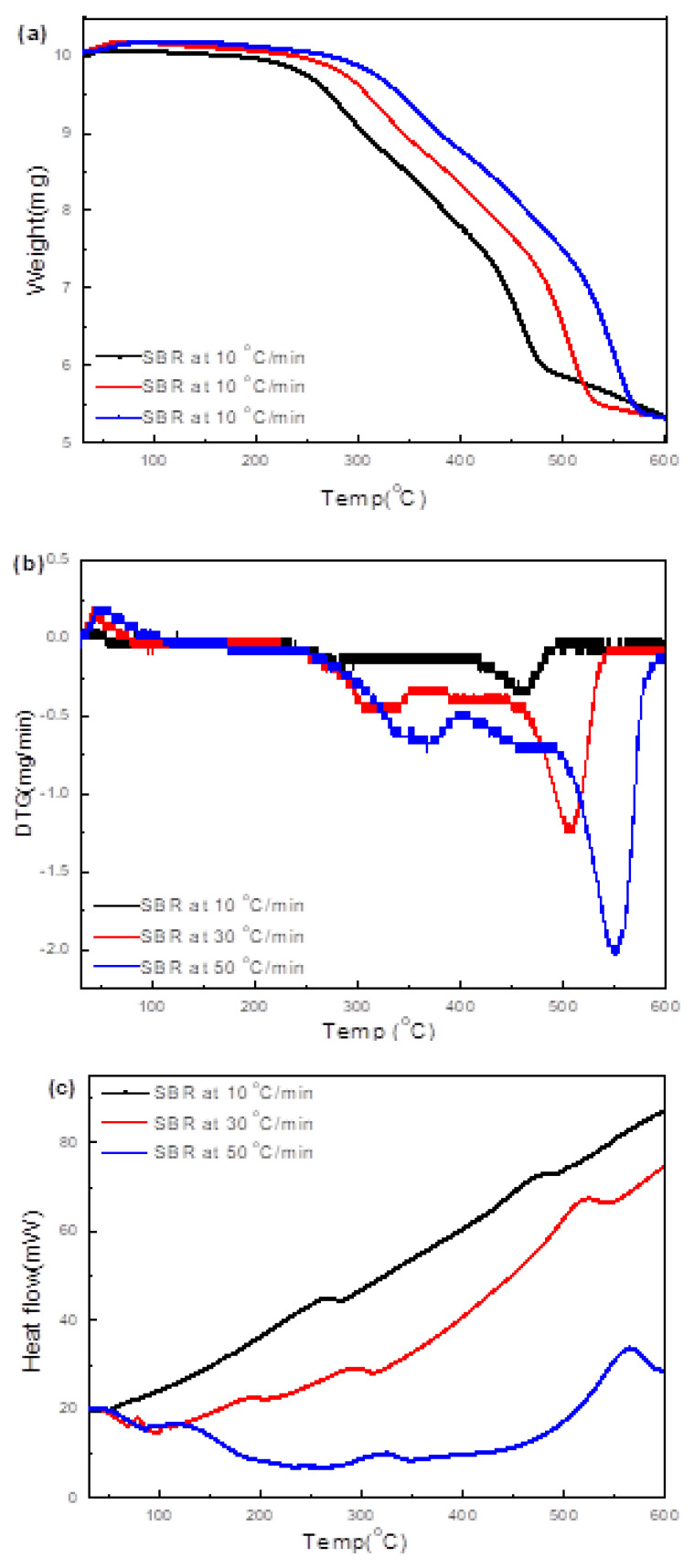

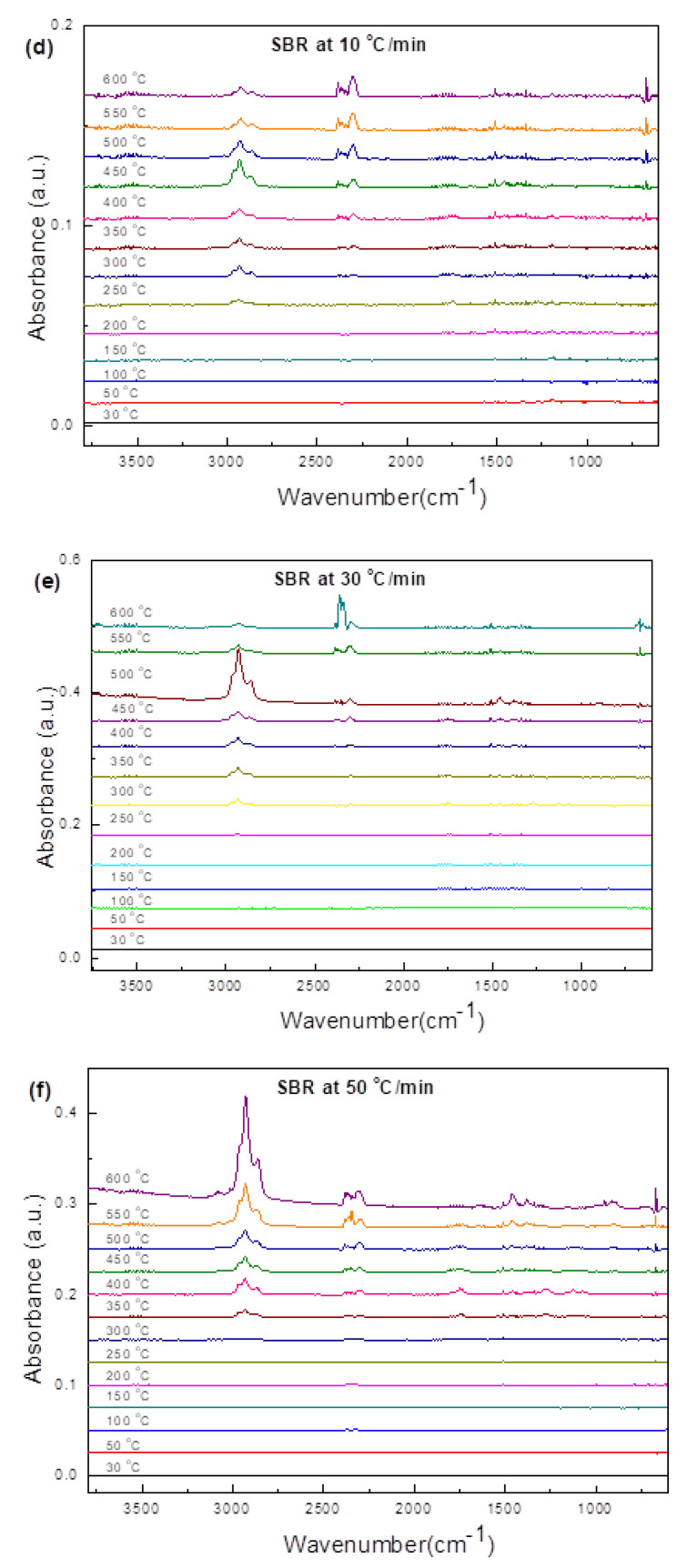
(**a**–**c**) Thermal analyses (TG/DTG/DSC) data of the SBR during heating at 10, 30 and 50 °C/min to 600 °C. (**d**–**f**) Corresponding evolved gas IR spectra of soft black rubber (SBR) during heating to different temperatures at the three rates.

TG/DTG/DSC plots of pyrolysis of soft black rubber (SBR) at 10, 30, and 50 °C/min, presented in Figure 12a–c, suggest the residual char at the three heating rates to be ~50%, which is broadly consistent with the residual char of 49% suggested by the TG/DTG data in Figure 4. Also consistent is the fact that the weight loss took place in two stages. The maximum transformation in the first stage occurred at ≥300 °C, whereas that in the second stage occurred at 450 °C. These transformations are attributed to the reduction of the oxygen-containing functional groups (e.g., carbonyl, hydroxyl, or carboxyl groups) to CO_2_, methane and H_2_O. At 50 °C/min, the CH_4_ evolution was prominent even at 600 °C, more than the CO_2_. Whereas CO_2_ evolution can limit the energy recovery (energy potential of the produced gas), the CH_4_ is a potential energy source (50–55 MJ/kg).

**Figure 13:**
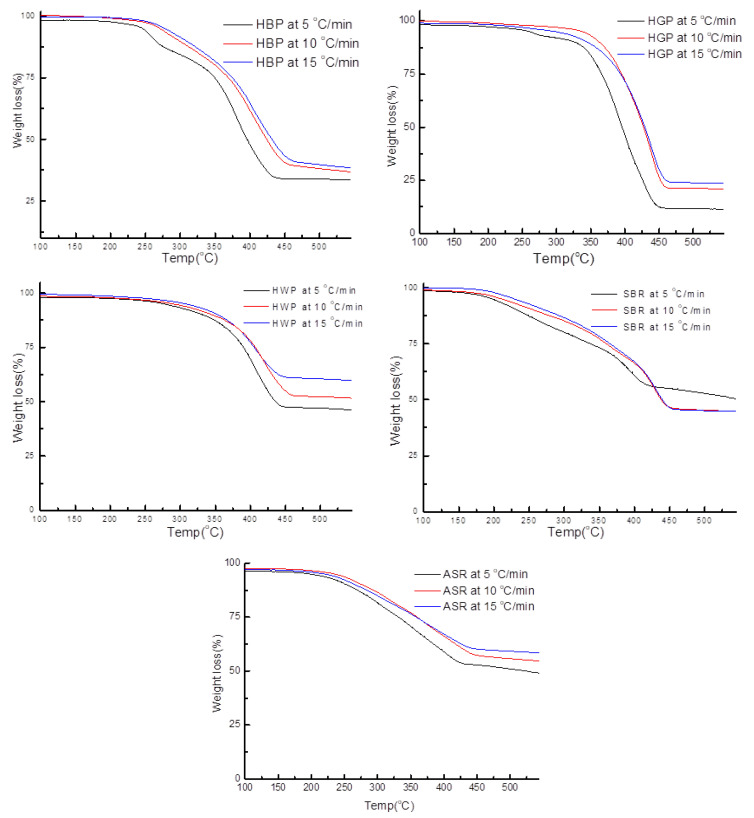

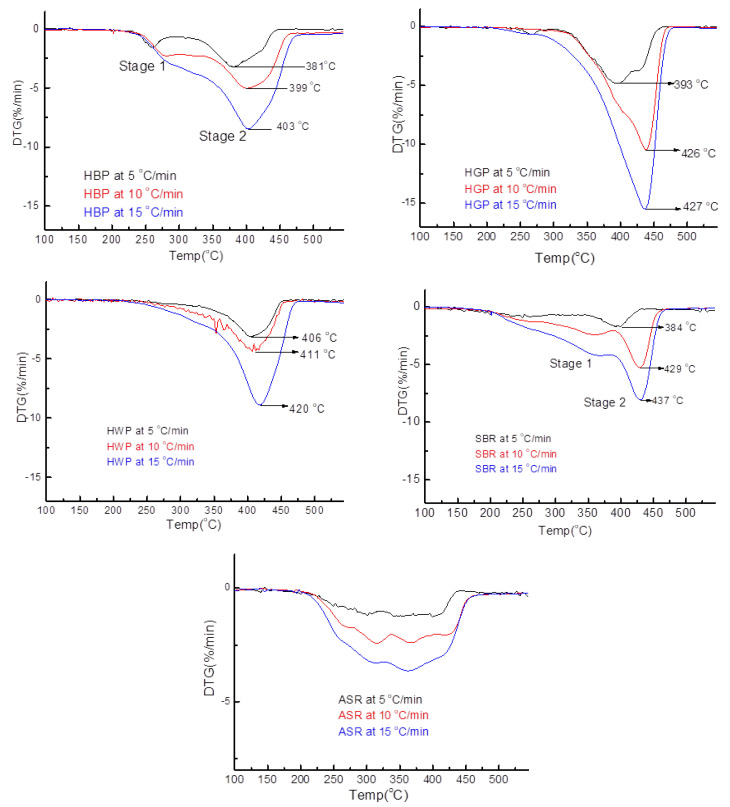
TG/DTG of the ASR components at the different heating rates at 5, 10, and 15 °C/min.

Figure 13 represents the TG/DTG curve for mixed ASR and its components, HBP, HGP, HWP, and SBR, at three different heating rates (5, 10, 15 °C/min). These results are also presented in Table A1. It was observed that the final residual weight was primarily dependent on the heating rates. In general, the residual weight was higher for higher heating rates, especially for HBP and HGP and HWP. The mixed ASR sample also showed a similar trend. At low heating rates, the time to which the sample was exposed to a given temperature was long enough for the material to equilibrate with the medium and therefore attain the temperature of the gas. At higher heating rates, the reaction mechanisms were dominated by the heat transfer and longer time for temperature equilibration within the sample, which may have delayed the breaking of bonds, and eventually, the release of volatiles.

**Figure 14:**
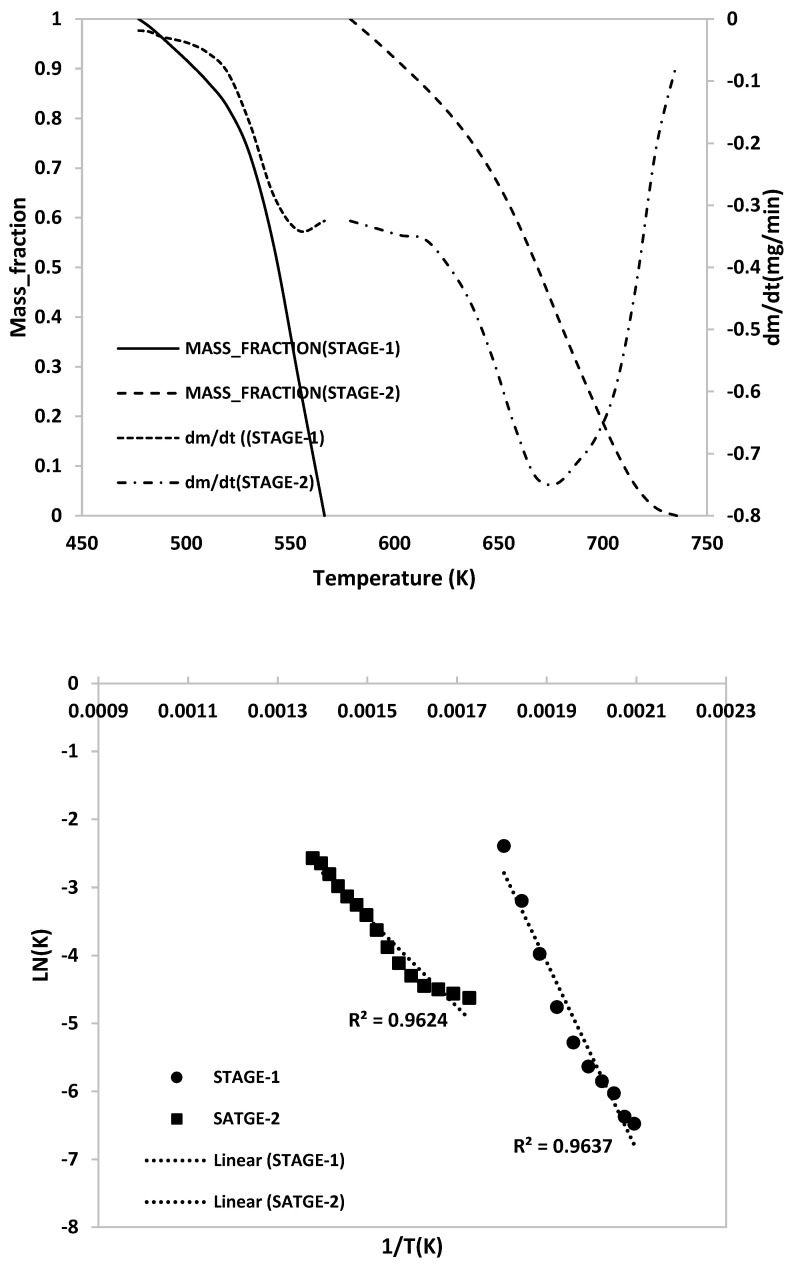
Thermal decomposition and Arrhenius plot of the HBP.

**Figure A1:**
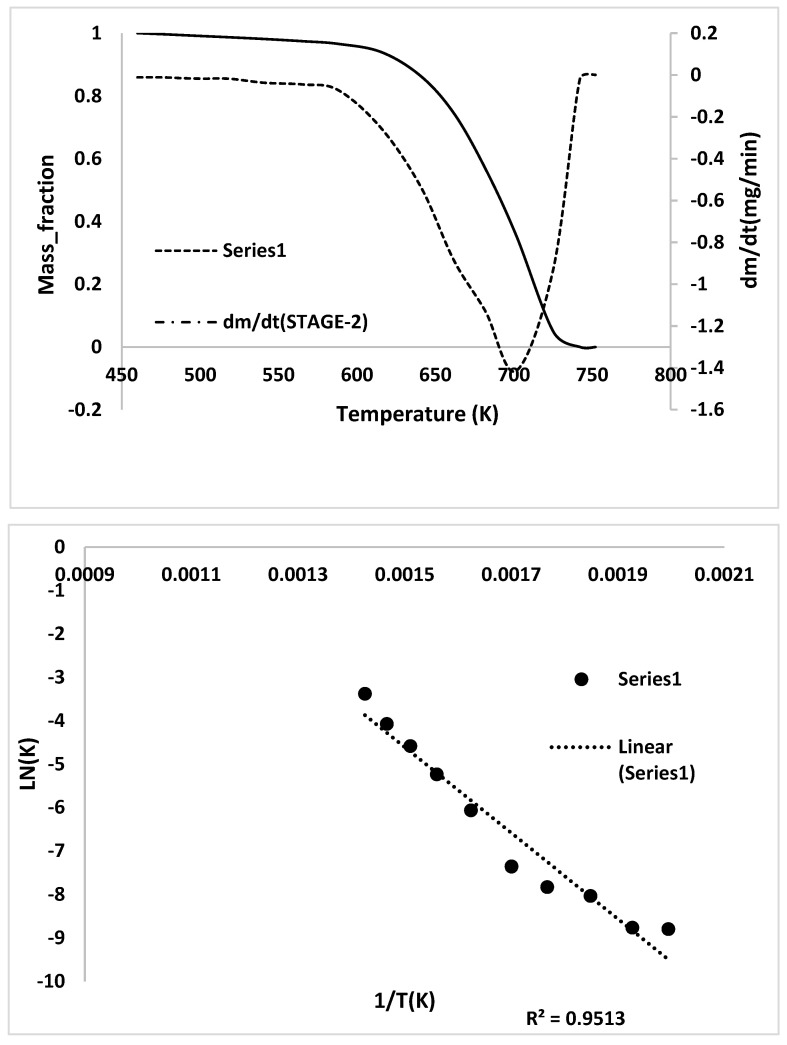
Thermal decomposition and Arrhenius plots of the HGP.

**Figure A2:**
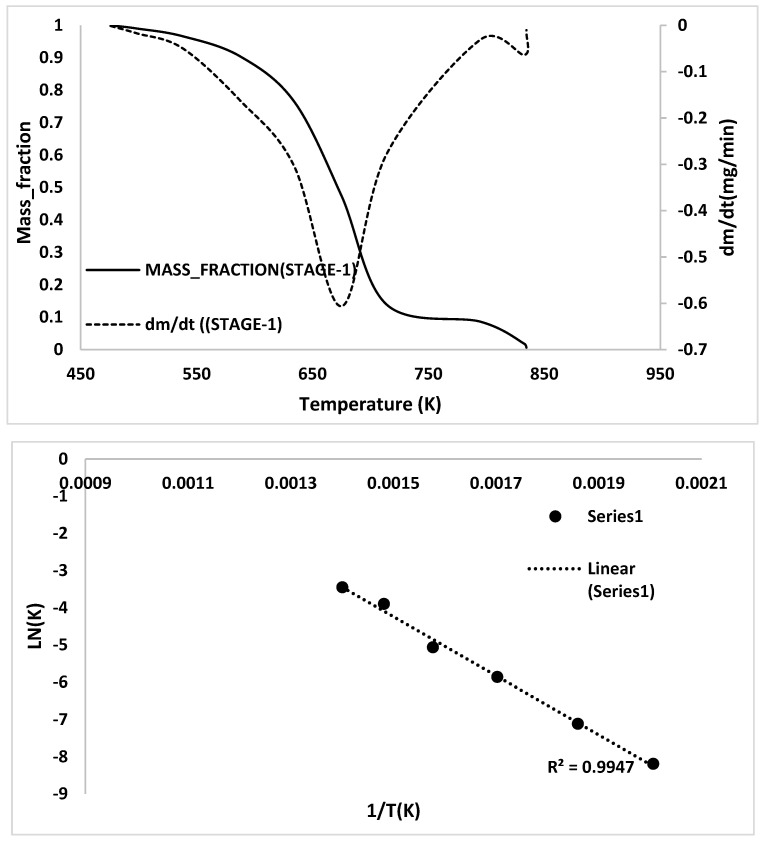
Thermal decomposition and Arrhenius plot of the HWP.

**Figure A3:**
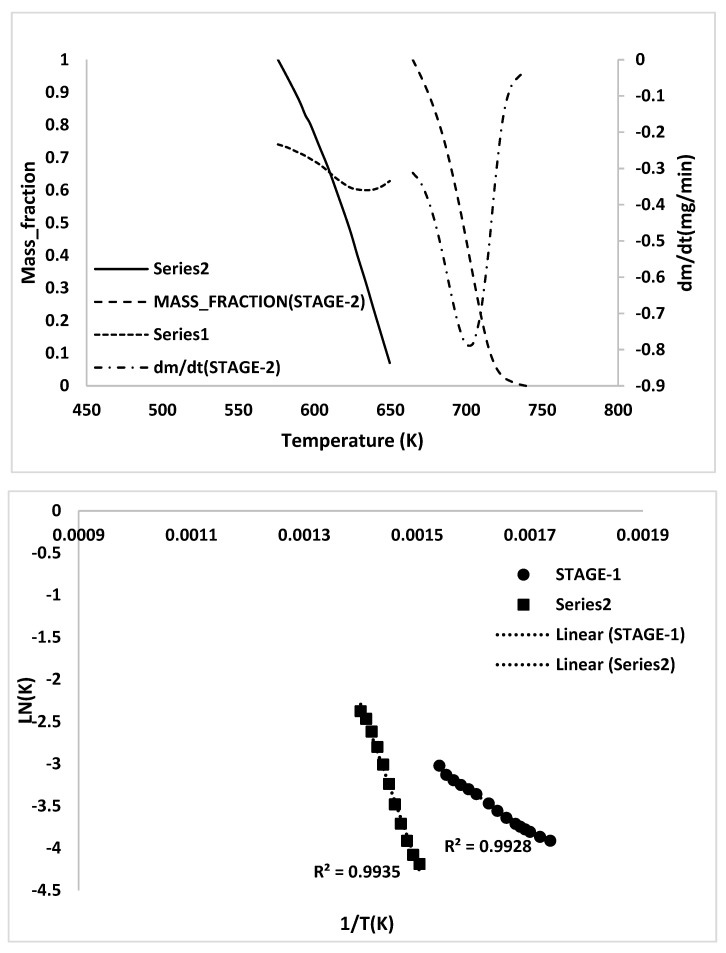
Thermal decomposition and Arrhenius plot of the SBR.

**Figure A4:**
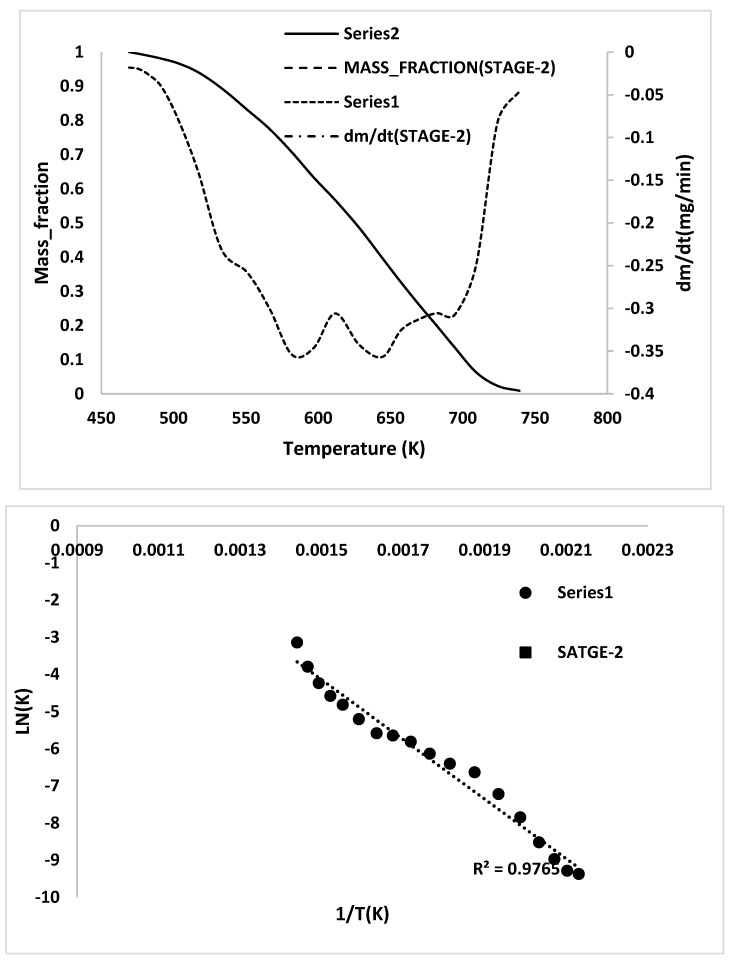
Thermal decomposition and Arrhenius plot of the ASR.

The kinetic analyses were performed employing the methods described in Section 2.3. The kinetic parameters for single and mixed ASR samples depended on the heating rates. The kinetic parameters for the ASR components were measured; Figure 14 represents the conversion curve of HBP samples at 10 °C/min between conversion X = (m_0_ − m)/(m_0_ − m_f_) and the conversion derivative. The reaction orders for each stage estimated using linear regression analysis showed higher coefficients. Although the data do not appear to be completely linear as observed, this was the closest fit we could achieve from the obtained data. The kinetic parameters at three heating rates of individual and mixed ASR components are presented in Table 8. As suggested in the table, a higher heating rate requires less activation energy for the decomposition during pyrolysis [24]. This is because at lower heating rates, surface/chemical control dominates, and at higher heating rates diffusion/mass transfer control dominate, characterised by lower activation energy (i.e., ~equal to heat of reaction). Similar results were reported by Yan et al. [24] for the degradation of waste and virgin PP and LDPE. The kinetic parameter profiles are provided in the Appendix Figure A1—HGP, Figure A2—HWP, Figure A3—SBR and Figure A4—ASR.

Reaction orders for all ASR component types was in the range of 0.1–1.8 in each stage for regression analysis, showing high correlation coefficients. The corresponding activation energies and frequency factors of each ASR component type were calculated to be 24.98–124.94 kJ/mol and 3.34 × 10^13^ to 5.68 × 10^1^ mg^−1^/min, respectively. Multistage linearization provided a very good fit (0.9513 < R^2^ < 0.9981) for all heating rates. Figure 14 represents the graphical representation of linearized data for HBP. The activation energies of all ASR components are listed in Table 8.

## 4. Conclusions

The pyrolysis of four different components of a given ASR (i.e., hard black plastic (HBP), hard grey plastic (HGP), hard white plastic (HWP), and soft black rubber (SBR)) was presented. The study included the investigation of the morphology, chemical analyses, thermal analyses, and simultaneous IR spectroscopy of the evolving gases. A novel characterisation method using in situ synchrotron IR was employed to observe the changes in functional groups of HBP, HGP, HWP. and SBR during heating to different temperatures at different heating rates.

(i) Morphology differs considerably among the different ASR component types and their chars, suggesting the difference in their chemical nature. The main matrix in each case was polymeric that pyrolysed with considerable char residue. There were considerable amounts of rod-shaped structures identified to be compounds such as oxides of Te, Sr, Si, As, Dy, Rn, Ti, and Nb (these thermally stable compounds do not transform during pyrolysis).
(ii) TG/DTG, during heating to different temperatures (up to a maximum of 600 °C) at different heating rates, suggested either a single- or two-stage pyrolysis for different ASR components.
(iii) Transformations in the first stage were generally attributed to the degradation of oxygen-containing functional groups, such as carbonyl, hydroxyl, or carboxyl groups (e.g., aldehydes and esters that are present in ASRs as impurities, additives, stabilizers, and/or oils) into carbon dioxide, methane, and water. Such transformations occurred at an elevated temperature, and the temperature range varied for HBP, HGP, HWP, and SBR.
(iv) The second stage transformations occurred at higher temperatures, probably due to the thermal degradation of the polymer chain.
(v) The TG/DTG/DSC and simultaneous gas-phase IR spectroscopy confirmed the evolution of at least some of the gases as described in (iii) above.
(vi) In industrial practice, one would like to speed up the pyrolysis of ASRs into useful products such as oil by employing high heating rates. However, the synchrotron IR data presented in this study clearly demonstrate that a high heating rate (such as 150 °C/min) results in an incomplete conversion of ASRs unless necessary time is provided.
(vii) To complement the information on the evolution of functional groups from ASR pyrolysis as a function of isothermal pyrolysis temperature and estimation of kinetic parameters based on multistage modelling, in situ transmission IR spectroscopy and TG analysis were used extensively. Thermogravimetric analysis shows that the maximum weight loss of ASR components occurred in the temperature range of 182–489 °C, at which 63.16, 82, 48.6, 51.76, 47.1% for the HBP, HGP, HWP, SBR, and the ASR mixture, respectively, was devolatilized. The linearized multistage kinetic model was applied to TGA data to obtain a good fit with a range (0.9513 < R^2^ < 0.9981) for all heating rates. Activation energy of different pyrolysis stages (24.98–124.94 kJ/mol) decreased with the increase in heating rate. The corresponding frequency factor ranged from 3.34 × 10^13^ to 5.68 × 10^1^ mg^−1^/min. The reaction order for different stages in decomposition was in the range from 0.1 to 1.8.

## Figures and Tables

**Table 1:** Proximate and ultimate analysis and calorific value of ASR.

Proximate Analysis (wt.%)						Ultimate Analysis (wt.%)					
Sample	T_max_ °C	VM	A	M	FC	C	H	N	S	O_diff_	GCV (MJ/kg)
HBP	290, 399	62.1	17.0	0.8	20.0	61.9	6.4	2.7	0.3	28.6	24.8
HGP	436	82.0	0.3	0.1	17.5	69.7	8.6	3.4	0.1	18.4	32.6
HWP	411	48.6	28.3	0.8	22.2	49.0	5.5	1.2	0.2	43.9	17.5
SBR	358, 429	51.7	23.5	4.3	20.3	65.0	7.2	0.5	0.8	26.4	27.6
ASR	162–475	47.1	35.5	3.6	13.8	-	-	-	-	-	-

Calculated by difference; HBP; hard black plastic; HGP: hard grey plastic; HWP: hard white plastic; SBR: soft black rubber; ASR: automotive shredder residue; T_max_; peak decomposition temperature (determined from the differential thermogravimetric analysis at heating rate of 10 °C/min; M: moisture; A: Ash, VM: volatile matter; FC: fixed carbon; S: sulphur; C: carbon; N: nitrogen; O: oxygen; GCV gross calorific value (MJ/kg).

**Table 2:** Most common plastic types in ASR [22].

Plastics	Wt.%
Polypropylene	39
Polyethylene	28
Polycarbonate	12
Polyurethane	5
other	16

**Table 3:** Decomposition temperature ranges of PE, PP, PS, NR, and SBR.

Polymer Type	Temp Range (°C)	Peak Temp (°C)	References
**HDPE (10 °C/min)**	350–380	480	[23]
**LDPE (10 °C/min)**	380–500	470	[24]
**PE (20 °C/min)**	400–520	480	[25]
**LDPE (20 °C/min)**	410–515	491	[26]
**PP (10 °C/min)**	328–410	464	[27]
**NR (10 °C/min)**	-	378	[28,29]
**SBR (10 °C/min)**	-	447	[28,29]
**BR (10 °C/min)**	-	467	[28,29]
**BR + SBR (10 °C/min)**	-	427	[28,29]

Note: HDPE—high-density polyethylene; LDPE—low-density polyethylene, PP—polypropylene; NR—natural rubber; SBR—styrene butadiene rubber; BR—butadiene rubber.

**Table 4:** Characteristic IR peaks expected and detected in the HBP at 10 and 150 °C/min.

10 °C/min, Current Data, Wave Number (cm^−1^)	150 °C/min, Current Data, Wave Number (cm^−1^)	Functional Groups	Wave Number (cm^−1^)	Reference
**3293**	**3300**	Amide N--H stretch	3550–3250	[32]
**2958**	**2980**	Methylene (as) C--H stretching	2936–2843	[32]
**2962**	**2918**	Methylene (as) C-H stretching	2936–2843	[32]
**2855**	**2800**	Methylene (s) C-H stretching, C--H stretch in N-CH_2_	2936–2843, 2820–2760	[32]
**1640**	**1640**	Olefinic -C=C-, Amide C=O stretching	1680–1600 1695–1630	[32]
**1534**	**-**	Amide II N-H bending and C-N stretching	1565–1508	[32]
**1452**	**1470**	CH2-CH_2_-[(NH-C-H bending	1475–1445	[32]
**1361**	**1382**	Methyl C-H bending	1390–1370	[32]
**-**	**1282**	Amide III C-N stretching -NH bending	1300–1000	[32]
**-**	**1233**	Amide III C-N stretching -NH bending	1300–1000	[32]
	**1150**	CC-H (s) bending/CH_2_ twisting	-	[32]
**996**	**999**	C-C stretching and bending	1000–800	[32]

**Table 5:** Characteristic IR peaks expected and detected in the HGP at 10 and 150 °C/min.

10 °C/min, Current Data, Wave Numbers (cm^−1^)	150 °C/min, Current Data, Wave Numbers (cm^−1^)	Functional Groups	Wave Number (cm^−1^)	References
**2954**	**2961**	Methyl (s) C-H stretching	2972–2862	[32,33,34]
**2914**	**2916**	Methylene (s) C-H stretching	2936–2843	[32,33,34]
**2875**	**2868**	Methyl (s) C-H stretching	2972–2862	[32,33,34]
**2848**	**2872**	Methyl (s) C-H stretching	2972–2862	[32,33,34]
**1456**	**1459**	Methylene C-H bending O-H bending	1475–1445 1440–1330	[32,33,34]
**1373**	**1377**	Methyl C-H bending O-H bending	1390–1370 1390–1310	[32,34,35]
**1160**	**1167–1043**	Methylene (s) C-H deformation C-O stretch	1250–900 1150–1085	[34]
**-**	**997**	C-C stretching, bending and rocking	1000–800	[32,33,36]
**1000**	**974**	C-C stretching, bending and rocking	1000–800	[32,33,36]

**Table 6:** Characteristic IR peaks expected and detected in the HWP at 10 and 150 °C/min.

10 °C/min, Current Data, Wave Numbers (cm^−1^)	150 °C/min, Current Data, Wave Numbers (cm^−1^)	Functional Group	Wave Number (cm^−1^)	References
**3067** **–** **3024**	**3061–2025**	Aromatic C-H stretching	3100–3000	[32,34]
**2935–2851**	**2938–2853**	Methylene (s) C-H stretching	2936–2843	
**1873–1734**	**1751–1701**	Overtone bands mono-substituted benzene rings	2000–1700	[34]
		Carbonyl C=O stretching	1760–1700	[32,34]
**1597** **–** **1490**	**1600–1495** **1530–1515**	Aromatic C=C stretching and methyl C--H bending Carbonyl C=O stretching	1600–1430	[32,33,34,37]
**1367**	**1305**	Methylene C-H wagging	1390–1370	[32,33,34]
**1188** **–** **1026**	**1139–1047**	Aromatic C-H bending	1250–900 1275–1000	[34]

**Table 7:** Characteristic IR peaks expected and detected in the SBR 10 and 150 °C/min.

150 °C/min, Current Data, Wave numbers (cm^−1^)	Functional Groups	Wave Number (cm^−1^)	References
**3310**	O-H stretching	3550–3200	[32]
**2972–2885**	Methylene (s) C-H stretching	2936–2843	[32]
**1733–1707**	Carbonyl C=O stretching	1760–1700	[32,34]
**1600**	C=C stretching	1600–1680	[32,34]
**1533–1515** **1454–1374** **1312–1100**	Carbonyl C=O stretching Methylene C-H bending and wagging Vinyl CCH bending and twisting	- 1470–1450 -	[34] [34] [34]

**Table 8:** Kinetic parameters for thermal decomposition of the ASR components.

**Hard black plastic (PA)** **5 °C/min**	***n***	1	*n*	0.3
	**E**	124.94	E	85.99
	**A**	3.34 × 10^13^	A	1.06 × 10^7^
**10 °C/min**	***n***	0.9	*n*	0.6
	**E**	114.52	E	54.73
	**A**	3.83 × 10^10^	A	6.20 × 10^3^
**15 °C/min**	***n***	0.3	*n*	0.6
	**E**	106.43	E	35.73
	**A**	1.19 × 10^10^	A	4.72 × 10^2^
**Hard grey plastic (PP)** **5 °C/min**	***n***	1.1	*n*	1
	**E**	76.20	E	126.47
	**A**	2.13 × 10^6^	A	1.00 × 10^9^
**10 °C/min**	***n***	1	*n*	N/A
	**E**	71.20	E	
	**A**	4.32 × 10^4^	A	
**15 °C/min**	***n***	0.1	*n*	N/A
	**E**	71.23	E	
	**A**	1.86 × 10^5^	A	
**Hard white plastic (PS)** **5 °C/min**	***n***	1.1	*n*	N/A
	**E**	67.00	E	
	**A**	3.52 × 10^11^	A	
**10 °C/min**	***n***	1	*n*	N/A
	**E**	59.64	E	
	**A**	2.14 × 10^4^	A	
**15 °C/min**	***n***	0.1	*n*	N/A
	**E**	51.16	E	
	**A**	5.20 × 10^3^	A	
**Soft black rubber (XNBR)** **5 °C/min**	***n***	0.1	*n*	0.8
	**E**	62.08	E	124.27
	**A**	3.77 × 10^2^	A	2.60 × 10^9^
**10 °C/min**	***n***	0.2	*n*	0.6
	**E**	35.77	E	110.11
	**A**	4.43 × 10^2^	A	3.25 × 10^11^
**15 °C/min**	***n***	0.1	*n*	0.6
	**E**	24.98	E	121.79
	**A**	5.68 × 10^1^	A	1.03 × 10^9^
**Mixed ASR** **5 °C/min**	***n***	1.7	*n*	N/A
	**E**	92.24	E	
	**A**	2.67 × 10^4^	A	
**10 °C/min**	***n***	1.7	*n*	N/A
	**E**	78.66	E	
	**A**	2.67 × 10^4^	A	
**15 °C/min**	***n***	1.8	*n*	N/A
	**E**	71.18	E	
	**A**	1.29 × 10^5^	A	

## Data Availability

All the data in the article are available within the article.

## Appendix E

**Table A1:** Characteristic temperature of pyrolysis of the ASR components at 5, 10, and 15 °C /min.

Plastics	Stage-1 Devolatilization	Stage-2 Devolatilization
**Hard black plastic (HDPE)** **5 °C/min**	Range: 212–293 °C Peak: 261 °C	Range: 312–455 °C Peak: 381 °C
**10 °C/min**	Range: 211–302 °C Peak: 290 °C	Range: 355–470 °C Peak: 399 °C
**15 °C/min**	Range: 213–309 °C Peak: 294 °C	Range: 332–482 °C Peak: 403 °C
**Hard grey plastic (PP)** **5 °C/min**	Range: 241–287 °C Peak: 267 °C	Range: 299–469 °C Peak: 393 °C
**10 °C/min**	Peak 1 Disappeared	Range: 230–481 °C Peak: 436 °C
**15 °C/min**	Peak 1 Disappeared	Range: 212–486 °C Peak: 437 °C
**Hard white plastic (PS)** **5 °C/min**	Range: 204–467 °C Peak: 406 °C	N/A
**10 °C/min**	Range: 243–468 °C Peak: 411 °C	
**15 °C/min**	Range: 246–489 °C Peak: 420 °C	
**Soft black rubber (NR & SBR)** **5 °C/min**	Range: 145–297 °C Peak: 247 °C	Range: 341–433 °C Peak: 394 °C
**10 °C/min**	Range: 148–386 °C Peak: 358 °C	Range: 391–478 °C Peak: 429 °C
**15 °C/min**	Range: 160–385 °C Peak: 367 °C	Range: 390–482 °C Peak: 432 °C
**Mixed ASR** **5 °C/min**	Range: 132–448 °C Peak: N/A	N/A
**10 °C/min**	Range: 162–475 °C Peak: N/A	
**15 °C/min**	Range: 166–478 °C Peak: N/A	
